# Association of rs9679162 Genetic Polymorphism and Aberrant Expression of Polypeptide *N-Acetylgalactosaminyltransferase 14 (GALNT14)* in Head and Neck Cancer

**DOI:** 10.3390/cancers14174217

**Published:** 2022-08-30

**Authors:** Nan-Chin Lin, Yin-Hwa Shih, Kuo-Chou Chiu, Po-Jung Li, Hui-Wu Yang, Wan-Chen Lan, Shih-Min Hsia, Tong-Hong Wang, Tzong-Ming Shieh

**Affiliations:** 1 Department of Oral and Maxillofacial Surgery, Show Chwan Memorial Hospital, Changhua 500009, Taiwan; 2 Department of Oral and Maxillofacial Surgery, Changhua Christian Hospital, Changhua 500, Taiwan; 3 Department of Healthcare Administration, Asia University, Taichung 41354, Taiwan; 4 Division of Oral Diagnosis and Family Dentistry, Tri-Service General Hospital, National Defense Medical Center, Taipei 11490, Taiwan; 5 School of Dentistry, China Medical University, Taichung 40402, Taiwan; 6 School of Nutrition and Health Sciences, Taipei Medical University, Taipei 110301, Taiwan; 7 Graduate Institute of Health Industry Technology and Research Center for Chinese Herbal Medicine, College of Human Ecology, Chang Gung University of Science and Technology, Taoyuan 33305, Taiwan; 8 Tissue Bank, Chang Gung Memorial Hospital, Taoyuan 33305, Taiwan; 9 Graduate Institute of Natural Products, Chang Gung University, Taoyuan 33305, Taiwan

**Keywords:** alcohol, betel nut, *GALNT14*, head and neck cancer, rs9679162, survival, polymorphism, prognosis, chemoradiotherapy

## Abstract

**Simple Summary:**

Neoadjuvant chemotherapy was performed before surgery. Because the tumor itself and the surrounding vascular bed were not damaged, the chemotherapy we performed could have good drug delivery. After the operation, the volume of the tumor can be reduced to facilitate surgery or radiotherapy. However, neoadjuvant chemotherapy also delays the patient’s time to receive main therapy. The physician must make sure that it has a good response and does not allow disease progression in the patient during neoadjuvant chemotherapy. Therefore, predicting the treatment response of neoadjuvant chemotherapy can shorten the treatment time, reduce the harm of chemotherapy side effects, and avoid the occurrence of drug resistance. The results of this study showed that *GALNT14*-rs9679162 and mRNA expression were associated with post-treatment survival in head and neck cancer. It can be used as an indicator to predict the treatment response of neoadjuvant chemotherapy.

**Abstract:**

The polypeptide *N-Acetylgalactosaminyltransferase 14 (GALNT14)* rs9679162 and mRNA expression were associated with treatment outcome in various cancers. However, the relation of *GALNT14* and head and neck cancer were nuclear. A total of 199 patients with head and neck squamous cell carcinoma (HNSCC) were collected in this study, including oral SCC (OSCC), oropharyngeal SCC (OPSCC), laryngeal SCC (LSCC), and others. The DNA and RNA of cancer tissues were extracted using the TRI Reagent method. The rs9679162 was analyzed using polymerase chain reaction (PCR) and sequencing methods in 199 DNA specimens, and the mRNA expression was analyzed using quantitative reverse transcription PCR (RT-qPCR) methods in 68 paired RNA specimens of non-cancerous matched tissues (NCMT) and tumor tissues. The results showed that the genotype of TT, TG, and GG appeared at 30%, 44%, and 26%, respectively. Non-TT genotype or G alleotype were associated with alcohol, betel nut, and cigarette using among patients with OSCC, and it also affected the treatment and survival of patients with OSCC and LSCC. High *GALNT14* mRNA expression levels increased lymphatic metastasis of patients with HNSCC, and treatment and survival in patients with OPSCC. Overall, the *GALNT14*-rs9679162 genotype and mRNA expression level can be used as indicators of HNSCC treatment prognosis.

## 1. Introduction

The N-acetylgalactosaminyltransferase (GALNT) enzyme family contains 20 members (GALNT1-20), which mediates protein O-glycosylation by transfering the N-acetyl-D-galactosamine (GalNAc) residue of UDP-GalNAc to the hydroxyl group of serines and threonines in target peptides [1]. GALNT6 has been shown to transfer GalNAc to large proteins such as mucins. Abnormal regulation of mucin-type O-glycosylation of proteins affects the malignancy of cancer cells, including tumor neogenesis, cell replication, migration, metastasis, and drug resistance [2]. Recent studies revealed that GALNT14, which is involved in various biological functions, has abnormal expression in various cancers [3]. Approximately 30% of the samples from various human malignancies show GALNT14 overexpression, and GALNT14 affects the O-glycosylation of death receptors in cancer cells and modulates sensitivity to cancer therapy [4]. *GALNT14* mRNA and protein are upregulated in the chemoresistant breast cancer cell line MCF7 [5]. *GALNT14* expression is upregulated and correlated with ovarian cancer [6]. Downregulation of *GALNT14* significantly inhibits apoptosis and ferroptosis in ovarian cancer cells [1].

*GALNT14* gene is located on chromosome 2, with 16 exons, and its mRNA is translated into 552 amino acids with a molecular mass of 64,321 Da. The single nucleotide polymorphism (SNP) *GALNT14* rs9679162 is located in intron 3, and the genotypes are TT, GT, and GG. Although this SNP does not affect the post-translational amino acid sequence, it is linked to cancer prognosis during chemotherapy. In advanced hepatocellular carcinoma (HCC) patients, the rs9679162 genotypes are associated with the objective response to chemotherapy using 5-fluorouracil, mitoxantrone, and cisplatin (FMP) [7] and with the outcome of chemoembolization plus sorafenib therapy [8]. HCC patients with the TT genotype have a significantly better median overall survival, time-to-progression, response rate, and disease control rate than HCC patients with non-TT genotypes [9,10]. Whereas the GG genotype is associated with a longer time and partial response to concurrent chemoradiotherapy (radiotherapy combined with FMP), in patients with esophageal squamous cell carcinoma [11]. In addition to HCC, the *GALNT14* SNP has been shown to predict progression-free survival (PFS), overall survival (OS), and response to chemotherapy in several types of gastrointestinal cancers, including cholangiocarcinoma, colorectal cancer, gastric cancer, esophageal cancer, and pancreatic ductal adenocarcinoma [12]. The *GALNT14* TT genotype is associated with unfavorable overall survival in patients with stage III colorectal cancer, receiving curative surgery and adjuvant oxaliplatin-based chemotherapy [13]. However, the GG genotype is associated with a significantly better overall survival than the non-GG genotypes in patients with resected pancreatic ductal adenocarcinoma [12].

Head and neck cancer develops from tissues in the oral cavity (mouth), pharynx, larynx (throat), paranasal sinuses, nasal cavity, salivary glands, nose, sinuses, and facial skin. The most common types of head and neck cancer occur in the lips, mouth, and larynx. Squamous cell carcinoma of the head and neck accounts for over 90% of head and neck cancers [14]. Head and neck squamous cell carcinoma (HNSCC) is the seventh most common type of cancer diagnosed worldwide, with more than 600,000 new cases diagnosed annually [15], and oral cancer is the most common HNSCC in North Eastern Nigeria, Yemen, and Taiwan [16,17,18]. Alcohol and/or tobacco are major risk factors for HNSCC. Chewing of betel nut is also a major risk factor for HNSCC in Taiwan and India. Approximately 70% of oropharyngeal cancers (including the tonsils, soft palate, and the base of the tongue) are linked to human papillomavirus (HPV) [19].

Traditionally, surgery and radiation therapy have been the treatments of choice for most types of head and neck cancers, and concurrent chemoradiotherapy improves the survival rates in HNSCC patients. The 5-year relative survival rate in head and neck cancers significantly improved from 54.7% in 1992–1996 to 65.9% in 2002–2006 [20]. Chemotherapy with modified docetaxel, cisplatin, and 5-fluorouracil (5-FU) (mTPF) is effective for the palliative treatment of recurrent and metastatic HNSCC in Asian patients [21]. However, some patients still have poor prognosis after mTPF treatment, which also causes unnecessary side effects. If the prognosis of patients after chemotherapy can be accurately predicted, better chemotherapy outcomes achieved and unnecessary side effects can be avoided. *GALNT14*-rs9679162 genotype is a predictor of PFS, OS, and response to FMP chemotherapy FMP in HCC, and *GALNT14* expression also affects chemoresistance in breast and ovarian cancer cells. However, the *GALNT14*-rs9679162 genotype and its expression in head and neck cancers have not been studied. Therefore, this study analyzed the frequency of the *GALNT14*-rs9679162 genotype and the expression level of *GALNT14* in patients with head and neck cancer. In addition, we investigated whether the *GALNT14*-rs9679162 genotype is related to GALNT14 mRNA expression.

## 2. Materials and Methods

### 2.1. Subjects

The study was conducted in accordance with the Declaration of Helsinki and approved by the Institutional Review Board (or Ethics Committee) of Changhua Christian Hospital (200501, 29 March 2022) for studies involving humans, and a total of 233 HNSCC cases were obtained from the Changhua Christian Hospital Tissue Bank. The tissue samples were immediately frozen in liquid nitrogen until further use. A selection process was performed on frozen sections to obtain HNSCC samples with more than 70% tumor cells, which were required for the analysis. The first diagnosis date was from 10 August 2007 to 16 September 2019. Samples with poor quality extracted DNA and RNA, those that failed PCR or RT-qPCR, and those with unclear sequencing signals were excluded. *GALNT14*-rs9679162 polymorphism analysis was performed in 199 HNSCC cases and *GALNT14* mRNA expression analysis was performed in 68 paired HNSCC and noncancerous matched tissues (NCMT). There were 62 cases of HNSCC overlapping in both the polymorphism and the mRNA expression analyses. The HNSCC cell lines A253, FaDu, HSC3, OEC-M1, SAS, and SCC9, and normal gingival epithelial SG cell lines were used for the in vitro study. The OSCC cell lines were kindly gifted by Professor Chi-Yuan Chen, (Chang Gung Memorial Hospital), and Professor Hsi-Feng Tu, (National Yang Ming Chiao Tung University). Cell culture conditions were as described previously [22].

### 2.2. DNA and RNA Extraction from Tissues

DNA and total RNA were extracted from various tissues using a TRI Reagent RNA isolation kit (Molecular Research Center, Cincinnati, OH, USA). After homogenizing the tissues in the TRI Reagent, 0.1 mL of 1-bromo-3-chloropropane or 0.2 mL of chloroform was added per ml of TRI Reagent used. The sample was covered tightly, shaken vigorously for 15 s, and allowed to stand for 2–15 min at room temperature. The resulting mixture was centrifuged at 12,000× *g* for 15 min at 2–8 °C. The DNA was in the phenol phase and interphase, and the RNA was in a clear hydrophilic layer. Further DNA and RNA separation and purification followed the manufacturer’s instructions. The quality and concentrations of DNA and RNA were measured by NanoVue Plus spectrophotometer (General Electric Company, Boston, MA, USA), and electrophoresis in 1% agarose gel. The samples were stored at −20 °C before use. The purified DNA and RNA were used for genotyping and gene expression assays, respectively.

### 2.3. Genotyping

For *GALNT14*-rs9679162 polymorphism analysis, 199 primary HNSCC cases, including 113 oral squamous cell carcinoma (OSCC) cases, 39 oropharyngeal squamous cell carcinoma (OPSCC) cases, 37 laryngeal squamous cell carcinoma (LSCC) cases, and 10 other SCC cases without previous treatment were included. DNA was isolated from these tissues using the TRI Reagent extraction method. *GALNT14* sequences containing the rs9679162 SNP were obtained by PCR using the following primers: Forward: 5′-TCACGAGGCCAACATTCTAG-3′, Reverse: 5′- TTAGATTCTGCATGGCTCAC-3′, with reaction conditions of 95 °C for 1 min, 58 °C for 1 min, and 72 °C for 1 min, for 40 cycles. Genotyping was performed by purifying the 172 bp PCR products from the gel using a Qiaex II Gel Extraction Kit, and sequencing them using a 377 DNA sequencer (Applied Biosystems, Foster City, CA, USA), according to the manufacturer’s instructions.

### 2.4. RT-qPCR

RT-PCR was performed as previously described [23]. All the RNA samples were treated with DNase I to remove the DNA contamination. For *GALNT14* mRNA analysis, 68 HNSCC and noncancerous matched tissue (NCMT) pairs were used, which included 27 paired oral cancer cases, 20 paired oropharynx cancer cases, 20 paired larynx cancer cases, and one paired laryngopharynx cancer case. A total of 62 HNSCC patients (25 paired oral cancer cases, 20 paired oropharynx cancer cases, 16 paired larynx cancer cases, and 1 paired laryngopharynx cancer case) overlapped in both the SNP genotyping and the mRNA expression analyses. *GALNT14* expression was analyzed by qPCR using the following primers: Forward: 5′-TAGCATCATCATCACCTTCCAC-3′, Reverse: 5′-TTACAGTCATCAGGGTCATTGC-3′ with reaction conditions of 95 °C for 30 s, 58 °C for 15 s, and 72 °C for 15 s. The specific PCR product was 141 bp [24]. *Glyceraldehyde-3-phosphate dehydrogenase* *(GAPDH)* served as an internal control (Forward: 5′-TGGTATCGTGGAAGGACTCATGAC-3′, Reverse: 5′-ATGCCAGTGAGCTTCCCGTTCAGC-3′). PowerUp SYBR Green Master Mix (Applied Biosystems, Waltham, MA, USA) was used for qPCR. Amplification of these genes was performed for 40 cycles at 95 °C for 15 s and 60–61.7 °C for 1 min, for 40 cycles. Three independent PCR reactions were performed to validate the reproducibility of the analysis. The Ct value of *GAPDH* was 20–30 in the NCMT and tumor tissues. Cases with inconsistent results were excluded from the final analysis. *GALNT14* expression in NCMT and HNSCC is shown as −∆Ct (Ct _GAPDH_–Ct _GALNT14_). *GALNT14* upregulation or downregulation in HNSCC is indicated by −∆∆Ct (−∆Ct _HNSCC_–∆Ct _NCMT_). The up- (−∆∆Ct ≥ 0) or downregulation (−∆∆Ct < 0) of *GALNT14* was used for receiver operating characteristic (ROC) analysis to determine the cut-off score.

### 2.5. Cytotoxicity Assay

The cytotoxicity assay was performed by seeding 5 × 10^3^ cells into each well of a 96-well cell culture plate. Cells were treated with vehicle alone or arecoline (12.5 and 100 µg/mL) for 24 h, docetaxel (6.25, 12.5, 25, 50, and 100 µg/mL), or 5-Fu (3.125, 6.25, 12.5, 25, 50, and 100 µg/mL) for 24, 48, and 72 h. Cell viability was assessed by performing a 3-(4,5-dimethylthiazol-2-yl)-2,5-diphenyltetrazolium bromide (MTT) assay, as described previously [25,26].

### 2.6. Western Blotting Analysis

Western blotting was performed using 50 µg of total protein from culture cells as described previously [27]. Depending on size, proteins were resolved on 7.5–12.5% polyacrylamide gel. The resolved proteins were transferred to 0.22 µm poly (vinylidene fluoride) (PVDF) membranes and blocked with 5% bovine serum albumin (BSA) for 1 h at room temperature (RT). The membranes were incubated with primary antibodies against GALNT14 (1:1000, sc-393051, SANTA CRUZ, Dallas, Texas, USA) and GAPDH (1:10,000, sc-32233, SANTA CRUZ) overnight at 4 °C. The membranes were then incubated with a peroxidase AffiniPure goat anti-mouse IgG secondary antibody (1:2000, 115-035-003, Jackson ImmunoResearch, Bar Harbor, ME, USA) for 2 h at RT, and visualized using the SuperSignal West Femto chemiluminescent substrate (Thermo Scientific, Rockford, IL, USA). GAPDH was used as an internal control. The results were quantitated using ImageJ software.

### 2.7. Statistical Analysis

Nonparametric analysis was performed, including the Mann–Whitney test for unpaired analysis and Wilcoxon signed-rank test for paired analysis. One-way ANOVA was used for comparing more than three groups. Fisher’s exact test, logistic regression, analysis of the odds ratio (OR), and 95% confidence interval (95% CI) were performed using Prism 9.0 (GraphPad Software, version 9.0.0, Irvine, CA., USA) or Statistical Package for the Social Sciences 12.0 (SPSS, Inc., Chicago, IL, USA). The survival curves were preformed using log-rank test. Differences between variants were considered significant at *p* < 0.05.

## 3. Results

### 3.1. Clinical Characteristics of the Subjects in GALNT14-rs9679162 Polymorphism Analysis

Subjects with incomplete medical records, PCR failures, or sequencing failures were excluded. A total of 199 HNSCC patients were included in this study, including 188 men and 11 women, with an average age of 57.33 ± 10.50 years. The subtypes of cancers include OSCC (113 cases), OPSCC (39 cases), LSCC (37 cases), and other cancers (10 cases). The top three types of head and neck cancer in Taiwan are oral, nasopharyngeal, and laryngeal cancers. Clinicopathological parameters—including subtype, age, sex, alcohol consumption, betel nut chewing, cigarette smoking habits, differentiation, tumor size, lymph node metastasis, AJCC 8th edition tumor stage, radiotherapy, chemotherapy, overall survival, and lesion site of oral cancer—are listed in Table S1. The case histories of some patients were missing, and some of them were categorized into stage BBB (no record). The most common primary sites in oral cancer subjects were the cheek and the tongue.

### 3.2. GALNT14-rs9679162 Genotype Frequency in HNSCC

**Figure 1:**
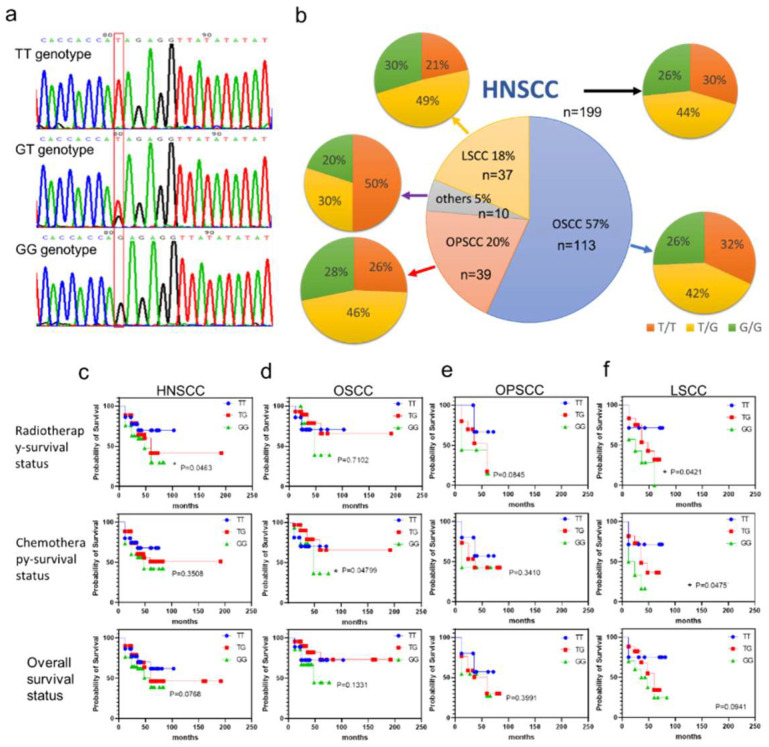
Relation between the *GALNT14*-rs9679162 genotype and radiotherapy, chemotherapy, and overall survival in HNSCC cases and its subtypes. *GALNT14*-rs9679162 sequencing and genotype frequency in HNSCC. (**a**) Direct sequencing of PCR products of *GALNT14*-rs9679162. The red box indicates TT, GT, and GG genotypes. (**b**) Genotype frequency of *GALNT14*-rs9679162 in 199 HNSCC, 113 OSCC, 39 OPSCC, 37 LSCC, and 10 other cancer subjects. Large pie chart; n, sample size in each type of cancer; percentage, percentage of each subtype of cancer in HNSCC. Small pie chart; orange, yellow, and green indicate the percentage of TT, TG, and GG genotypes in each type of cancer. Survival curves of patients exhibiting TT, TG and GG genotypes in (**c**) HNSCC, (**d**) OSCC, (**e**) OPSCC, and (**f**) LSCC. Log-rank test; * *p* < 0.05.

The HNSCC cases include OSCC (57%), OPSCC (20%), LSCC (18%), and other cancers (5%). The *GALNT14*-rs9679162 fragment was amplified by PCR followed by direct sequencing. The base of *GALNT14*-rs9679162 position is marked in the red box (Figure 1a). The single red peak and single black peak mean homologous TT genotype and GG genotype, respectively. One red and one black peak indicate a heterozygous TG genotype. Based on the sequencing data, the genotypes were identified as TT, GT, or GG (Figure 1a, red box). Among the 199 HNSCC cases, 59 cases were type TT (30%), 87 cases were type GT (44%), and 53 cases were type GG (26%). When all HNSCC samples were analyzed together, some clinical significance was ignored because of differences between the OSCC, OPSCC, and LSCC groups. The ratios of TT, TG, and GG genotypes within OSCC, OPSCC, LSCC, and other cancers are shown in Figure 1b. No significant differences in genotypic and allelic frequencies for *GALNT14*-rs9679162 were observed between patients with HNSCC and other cancer subtypes. The genotypic distribution of *GALNT14*-rs9679162 in HNSCC, OSCC, OPSCC, and LSCC patients did not deviate from the Hardy–Weinberg equilibrium (Figure 1b).

### 3.3. Comparison of Clinical Parameters between GALNT14-rs9679162 TT and Non-TT Genotypes in HNSCC

The association between *GALNT14*-rs9679162 genotypes and clinicopathological features was analyzed using binary logistic regression and showing p value, OR, and 95% CI in Table 1. The distribution of non-TT genotype (GT and GG genotypes) was associated with treatment-survival status in HNSCC and with risk factors in patients with OSCC. The frequency of the non-TT genotype was significantly higher in the HNSCC patients who died after radiotherapy, and in OSCC patients who consumed alcohol, chewed betel nut, and smoked cigarettes.

### 3.4. Alleotypes of GALNT14-rs9679162 in HNSCC

The association between *GALNT14*-rs9679162 alleles and clinicopathological features was analyzed using binary logistic regression analysis, and the p value, OR, and 95% CI are shown in Table 2. The G allele distribution was associated with treatment survival in HNSCC and LSCC patients, and risk factors in OSCC patients. The frequency of the G allele was significantly higher in HNSCC patients who died following radiotherapy, chemotherapy, in the betel nut and cigarette use groups in OSCC patients, and in the LSCC patients who died after radiotherapy and chemotherapy. The G allele frequency was higher in the OPSCC patients who died after radiotherapy as well. However, this difference was borderline (*p* = 0.0520; OR = 3.341) and approaching statistical significance.

### 3.5. Association between GALNT14-rs9679162 Genotype and Survival Rate in HNSCC Subjects

The association between *GALNT14*-rs9679162 genotypes and the survival status following radiotherapy, chemotherapy, and the overall survival status was investigated. The survival probability of *GALNT14*-rs9679162 GG genotype was significantly lower in the radiotherapy group (Figure 1c) of HNSCC patients, in the chemotherapy group of OSCC patients (Figure 1d), and in the radiotherapy and chemotherapy groups of LSCC patients (Figure 1f). However, the survival probability of *GALNT14*-rs9679162 GG genotype was not significantly different in OPSCC patients (Figure 1e).

### 3.6. Clinical Characteristics of the Subjects in GALNT14 mRNA Expression Analysis

A total of 68 HNSCC and noncancerous matched tissue (NCMT) pairs were included in this analysis, including 65 males and 3 females, with an average age of 57.85 ± 10.69 years. These included 27 oral cancer and NCMT pairs, 20 oropharynx cancer and NCMT pairs, 20 laryngeal cancer and NCMT pairs, and 1 laryngopharyngeal cancer and NCMT pair. The clinicopathological parameters of overall HNSCC and subtype cancer cases are shown in Table S2.

### 3.7. GALNT14 mRNA Expression in HNSCC and Its Subtypes

**Figure 2:**
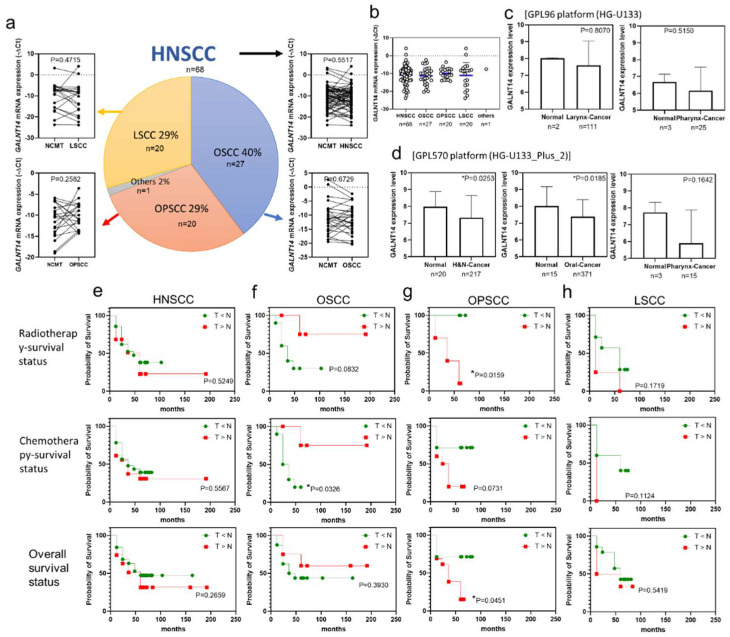
Relation between *GALNT14* mRNA expression (−∆Ct) trend and radiotherapy, chemotherapy, and overall survival in HNSCC and its subtypes. (**a**) 68 NCMT and HNSCC (include 27 OSCC, 20 OPSCC, 20 LSCC, and 1 other cancer subject) paired tissues were analyzed by RT-qPCR. Paired *t*-test was used to analyze the data. (**b**) *GALNT14* expression level (−∆Ct) in HNSCC, OSCC, OPSCC, LSCC, and other cancers. One-way ANOVA was used to analyze the data. (**c**) *GALNT14* expression level in larynx normal and cancer tissues. (**d**) *GALNT14* expression level in head and neck normal and cancer tissues, oral normal and cancer tissues, and pharynx normal and cancer tissues. n, sample size. For (**c**,**d**), Mann–Whitney test was used to analyze the data. * *p* < 0.05. The data generated in this analysis are publicly available in GENT2 at GPL95 platform (HG-133) and GPL570 platform (HG-133-Plus-2). Survival curves of patients with lower (T < N) and higher (T > N) *GALNT14* mRNA expression in tumor (T) compared to NCMT (N). (**e**) HNSCC, (**f**) OSCC, (**g**) OPSCC, and (**h**) LSCC. Log-rank test; * *p* < 0.05.

This analysis included cancer and NCMT pairs from OSCC (27 cases, 40%), OPSCC (20 pairs 29%), LSCC (20 pairs, 29%), and other cancers (1 pair, 2%). The *GALNT14* mRNA was reverse-transcribed and a 141 bp fragment was amplified using RT-qPCR, and the *GALNT14* expression level is shown as −∆Ct. In Figure 2a, the symbols and lines show the expression levels of *GALNT14* in each of the paired NCMT and cancer tissues. A slope of less than 0 indicates that *GALNT14* mRNA expression is lower in cancer (T < N), and a slope greater than 0 indicates the opposite (T > N). *GALNT14* mRNA expression was not significantly different in HNSCC, OSCC, OPSCC, and LSCC tissues compared to paired NCMTs (Figure 2a). It was also not significantly different among various cancer subtypes within HNSCC (Figure 2b). Analysis of the data from the GPL96 platform (HG-U133) from the Gene Expression Database of Normal and Tumor Tissues (GENT2) [28] revealed that the expression of *GALNT14* mRNA was not different in laryngeal and pharynx cancer tissues compared to that in unpaired normal tissues (Figure 2c). Analysis of the data from the GPL570 platform (HG-U133 plus 2) showed that the expression of *GALNT14* mRNA was also not different in pharyngeal cancer tissues, but was lower in head and neck cancer and oral cancer tissues compared to that in the unpaired normal tissues (Figure 2d). However, the results from the GENT2 database were different from those of our HNSCC subjects (Figure 2a). It is possible that the expression of *GALNT14* mRNA in NCMTs and normal tissues was different. The carcinogenic risk factors for HNSCC also differed between the GENT2 subjects and the subjects in this study.

### 3.8. Association between GALNT14 Expression and Clinical Parameters in HNSCC

We compared *GALNT14* expression in 68 paired NCMT and HNSCC tissue samples. The mean age of the 68 patients from whom the paired tissue samples were obtained was 57 years. Patients whose clinical information was partially lost or those who were categorized as stage BBB were excluded. The association between *GALNT14* mRNA expression levels and clinicopathological features was analyzed using binary logistic regression analysis, and the *p* value, OR, and 95% CI are shown in Table 3. The frequency of *GALNT14* upregulation was higher in the tumor tissues in the HNSCC patients showing lymphoid metastasis (subjects with N > 0 in TNM, N staging shown in Table 3). Similarly, the frequency of *GALNT14* upregulation was higher in the tumor tissues in the OPSCC patients who died after radiotherapy, or in the overall survival group. OPSCC patients who died after chemotherapy also showed a similar pattern, with the difference approaching significance. Though *GALNT14* expression was not associated with any other clinical parameters in OSCC and LSCC subjects, it was significantly different between OSCC and OPSCC subjects. *GALNT14* expression was not affected by alcohol, betel nut, or cigarette usage.

### 3.9. Correlation between GALNT14 Expression and Survival Rate in HNSCC

Of the 68 patients with HNSCC, 43 received radiotherapy, 15 did not; 44 received chemotherapy, 16 did not; and 36 received chemoradiotherapy. The radiotherapy survival curve, chemotherapy survival curve, and overall survival curve of HNSCC patients were analyzed using the log-rank test. The survival of OSCC patients following chemotherapy survival of patients with OSCC and the survival of OPSCC patients following radiotherapy and the overall survival of patients with OPSCC were significantly different between the *GALNT14* downregulation (N > T) and upregulation (N < T) groups. The *GALNT14* upregulation group showed a better chemotherapy survival rate than the GALNT14 downregulation group in OSCC patients (Figure 2f). However, the *GALNT14* downregulation group showed a better radiotherapy survival rate and overall survival rate than the upregulation group in patients with OPSCC (Figure 2g). The survival curves were not significantly different between the *GALNT14* downregulation and upregulation groups in HNSCC and LSCC patients (Figure 2e,h).

### 3.10. Correlation between GALNT14-rs9679162 Genotypes and GALNT14 mRNA Expression

**Figure 3:**
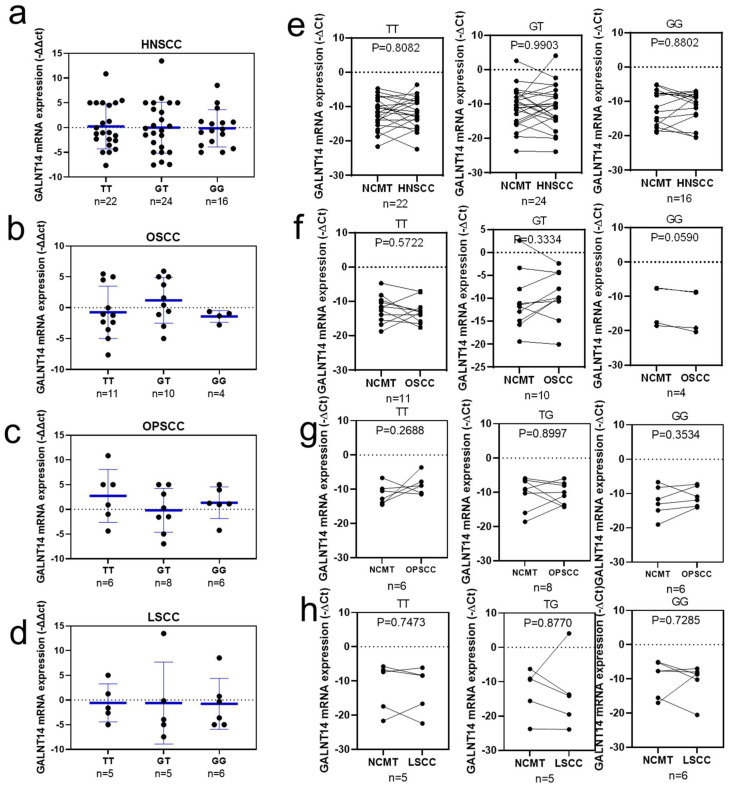
*GALNT14* mRNA expression levels of *GALNT 14*-rs9679162 genotypes in HNSCC, OSCC, OPSCC, and LSCC. *GALNT14* mRNA expression level (circle, −∆∆Ct of each paired tissue; blue lines, mean ± SD) in the three *GALNT14*-rs9679162 genotypes in (**a**) HNSCC, (**b**) OSCC, (**c**) OPSCC, and (**d**) LSCC. *GALNT14* mRNA upregulation or downregulation (circle, −∆Ct of each tissue) trend with respect to *GALNT14*-rs9679162 genotypes in paired NCMT and (**e**) HNSCC, (**f**) OSCC, (**g**) OPSCC, and (**h**) LSCC samples. n, sample size. One-way ANOVA was used to analyze the data in (**a**–**d**), and paired *t*-test was used for (**e**–**h**).

To clarify whether *GALNT14* mRNA expression levels are affected by *GALNT14*-rs9679162 genotypes, the results of *GALNT14* genotype and mRNA expression were combined and compared. Genotypes and mRNA expression were detected in 62 HNSCC cases, including 25 OSCC, 20 OPSCC, 16 LSCC, and 1 other cancer cases. There was no significant difference in *GALNT14* mRNA expression levels between HNSCC and OSCC, OPSCC, or LSCC. *GALNT 14*-rs9679162 genotype was not correlated with *GALNT14* mRNA expression levels (−∆∆Ct) in HNSCC (Figure 3a), OSCC (Figure 3b), OPSCC (Figure 3c), and LSCC (Figure 3d). *GALNT14* mRNA upregulation or downregulation (−∆Ct) trend related in various *GALNT 14*-rs9679162 genotypes was not significantly different in HNSCC, OSCC, OPSCC, or LSCC tissues, when compared to their respective NCMTs (Figure 3e–h, respectively). No relationship between *GALNT14*-rs9679162 genotype and *GALNT14* mRNA expression was found in the clinical data.

### 3.11. Relation between OSCC Risk Factors and GALNT14-rs9679162 Genotype or GALNT14 mRNA Expression in OSCC

**Figure 4:**
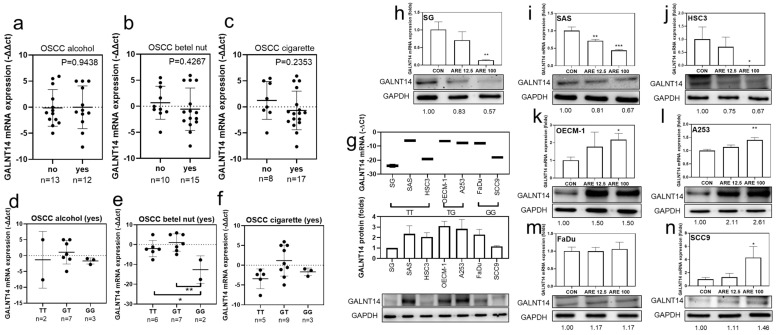
Relation between OSCC risk factors and *GALNT14*-rs9679162 genotype or *GALNT14* mRNA expression in OSCC. (**a**) *GALNT14* mRNA expression levels (circle, −∆∆Ct of each paired tissue; black lines, mean ± SD) with respect to alcohol consumption, (**b**) betel nut chewing, and (**c**) cigarette smoking in OSCC patients. (**d**) *GALNT14* mRNA expression in the *GALNT14*-rs9679162 genotypes in alcohol drinking, (**e**) betel nut chewing, (**f**) cigarette smoking OSCC patients. (**g**) *GALNT14* mRNA (−∆Ct, mean ± SD) and protein expression (folds) levels in seven OSCC cell lines. *GALNT14* mRNA and protein expression after treatment with vehicle, 12.5 or 100 μg/mL arecoline for 24 h in (**h**) SG, (**i**) SAS, (**j**) HSC3, (**k**) OECM-1, (**l**) A253, (**m**) FaDu, and (**n**) SCC9 cell lines. One-way ANOVA was used to analyze the data in (**d**–**n**). * *p* < 0.05, ** *p* < 0.01, *** *p* < 0.001.

GALNT14-rs9679162 non-TT genotypes increased alcohol, betel nut, and cigarette use (Table 1). The frequency of *GALNT14* mRNA downregulation in OSCC cases was higher than OPSCC cases (Table 3). *GALNT14* mRNA expression levels were not significantly different between alcohol, betel nut and cigarette users and non-users in the OSCC group (Figure 4a–c). However, 7 of the 12 alcohol users (58.33%), 10 of the 15 betel nut users (66.67%), and 12 of the 17 cigarette users (70.59%) showed a downregulation of *GALNT14* mRNA expression (−∆∆Ct < 0). There was no relationship between *GALNT14* mRNA expression levels and genotypes in alcohol and cigarette users (Figure 4d–f). Seven oral cancer cell lines were used to analyze GALNT14 expression levels in the three *GALNT14*-rs9679162 genotypes. SG, SAS, and HSC3 cell lines had GG genotype, OECM-1 and A253 cell lines had GT genotype, and FaDu and SCC9 cell lines had GG genotype. The top two cell lines with high GALNT14 expression were OECM-1 and A253 (Figure 4g). These data indicated that the actual *GALNT14* mRNA and protein expression levels were not correlated with the GALNT 14-rs9679162 genotype. The majority of the HNSCC patients used alcohol, betel nut, and cigarettes in combination. Hence, in order to reduce the confounding effects of the other variable factors, and to examine the specific effects of betel nut, we used arecoline in the following experiment. After 24 h treatment with arecoline treatment, GALNT14 mRNA and protein expression were downregulated in the SG, SAS, and HSC3 cell lines (Figure 4h–j); upregulated in the OECM-1, A253, and SCC9 cell lines (Figure 4k,l,n); and no significant change was observed in FaDu (Figure 4m). The results showed that *GALNT14* mRNA and protein expressions might be inhibited in the TT genotype, but was enhanced in non-TT genotypes after arecoline treatment in OSCC cell lines. Full pictures of the Western blots and the densitometry scans are presented in Figure S1.

### 3.12. Relationship between Chemoresistance and GALNT14-rs9679162 Genotype or GALNT14 mRNA Expression in OSCC

**Figure 5:**
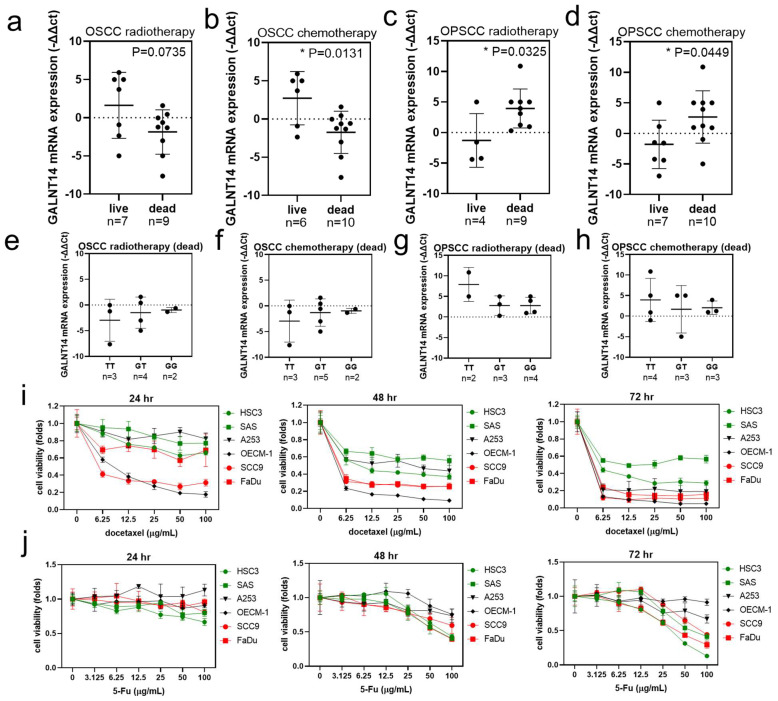
Relationship between *GALNT14* mRNA expression level, *GALNT14*-rs9679162 genotypes and radiotherapy, chemotherapy survival status in OSCC and OPSCC. *GALNT14* mRNA expression level (circle, −∆∆Ct of each paired tissue; black lines, mean ± SD) with respect to radiotherapy survival status in (**a**) OSCC and (**c**) OPSCC, and chemotherapy survival status in (**b**) OSCC and (**d**) OPSCC. *GALNT14* mRNA expression level (circle, −∆∆Ct of each paired tissue; black lines, mean ± SD) in the *GALNT14*-rs9679162 genotypes in the dead radiotherapy recipients in (**e**) OSCC and (**g**) OPSCC, and in the dead chemotherapy recipients in (**f**) OSCC and (**h**) OPSCC. (**i**) The chemoresistance ability of six OSCC cell lines after treatment with vehicle or 6.25–100 μg/mL docetaxel for 24, 48, and 72 h. (**j**) The chemoresistance ability of 6 OSCC cell lines after treatment with vehicle or 3.125–100 μg/mL 5-Fu for 24, 48, and 72 h. One-way ANOVA was used to analyze the data in (**b**–**h**). * *p* < 0.05.

*GALNT14*-rs9679162 genotype, allele and *GALNT14* mRNA expression seem to affect radiotherapy, chemotherapy, and overall survival status in OSCC and OPSCC. Hence, we re-analyzed the relationship between *GALNT14*-rs9679162 genotypes and *GALNT14* mRNA in patients with OSCC and OPSCC, who received radiotherapy and chemotherapy. The *GALNT14* mRNA expression was not significantly different between the live and dead radiotherapy recipients in the OSCC group (Figure 5a). However, the mRNA expression was significantly lower in the dead chemotherapy recipients in the OSCC group, compared to that in the live ones (Figure 5b). The *GALNT14* mRNA expression was higher in the dead radiotherapy and chemotherapy recipients in the OPSCC group, compared to that in the live ones (Figure 5c,d). However, the *GALNT14* mRNA expression level was not significantly different between the *GALNT14*-rs9679162 genotypes within the dead radiotherapy (Figure 5e,g) and chemotherapy recipient groups (Figure 5f,h) in OSCC and OPSCC groups. Cytotoxicity analysis of these cell lines showed that HSC3 and SAS cells were more tolerant to docetaxel (Figure 5i) and OECM-1 and A253 were more tolerant to 5-Fu compared to other OSCC cell lines (Figure 5j).

## 4. Discussion

According to the database of single nucleotide polymorphisms (dbSNP) [29], the distribution of *GALNT14*-rs9679162 T:G alleles was 56%:44% in Americans; 37%:63% in Europeans; 66%:34% in Africans; 55%:45% in East Asians; and 70%:30% in Japanese, indicating that *GALNT14*-rs9679162 distribution frequency was different among different races. In this study, the T allele accounted for 51.5% and the G allele accounted for 48.5%, which was close to the data on East Asians in the dbSNP database. The non-TT genotype is associated with poor survival and chemotherapy response in HCC and gastrointestinal cancers, but is beneficial for colorectal cancer and pancreatic ductal adenocarcinoma survival and chemotherapy [7,8,9,10,11,12,13]. Our results showed that the non-TT genotype frequency was higher in the dead HNSCC patients who received chemotherapy (Table 1), the GG genotype shortened the radiotherapy survival time (Figure 1c), and the G allele frequency was higher in the dead patients in the radiotherapy, chemotherapy, and overall survival groups (Table 2). Thus, the *GALNT14*-rs9679162 genotype is indicative of the therapeutic survival status of patients with HNSCC (Table 1 and Table 2, and Figure 1c). HNSCC includes OSCC, OPSCC, and LSCC according to the site of the primary tumor. Statistics showed that in OSCC, the non-TT genotype was associated with alcohol, betel nut, and tobacco use (Table 1); the G allele was associated with betel nut and tobacco use (Table 2); and the GG genotype shortened chemotherapy survival time (Figure 1d). The G allele was associated with death in radiotherapy recipients in OPSCC (Table 2). The G allele was associated with death in the radiotherapy and chemotherapy groups (Table 2), and the GG genotype shortened the radiotherapy and chemotherapy survival time in LSCC cases (Figure 1f). Therefore, the survival rate was poor in HNSCC patients with the *GALNT14*-rs9679162 non-TT genotype or the G allele, and the survival time of HNSCC patients with the GG genotype was short.

GALNT2 enhances the invasiveness of OSCC cells by modifying the O-glycans on epidermal growth factor receptor (EGFR) [30]. GALNT14 mediates the initial step of mucin-type O-glycosylation, and extensive O-glycosylation of mucin 1 (MUC1) contributes to cell resistance to anoikis [24]. High *GALNT14* mRNA expression might enhance the O-glycosylation of MUC1 or EGFR to promote lymphatic metastasis in HNSCC (Table 3). Although the NCMT and tumor tissues were all exposed to the same carcinogenic initiators and promoters, *GALNT14* downregulation in the OSCC tissues compared to paired NCMT were consistent with the lower expression of GALNT14 in oral cancer tissues compared to normal oral tissues in the public GENT2 profile analysis (Figure 2c,d). GALNT14 expression was not affected by alcohol, betel nut, or cigarettes in this study (Table 3). This may be because most of the HNSCC patients had more than one of these risk habits and different risk factor combinations interfere with GALNT14 expression. In Xena Functional Genomics Explorer (TCGA) [31] at GDC TCGA and TCGA Head and Neck Cancer profiles, *GALNT14* expression was induced by alcohol consumption (Figure S2a,d) and upregulated in the late stage of tumors (Figure S2b,e). The survival time of last 25% *GALNT14* expression group was significantly longer than top 25% GALNT14 expression group (Figure S2c,f). Therefore, *GALNT14* might be upregulated by alcohol consumption, but downregulated by betel nut chewing in *GALNT14*-rs9679162 TT genotype patients (Figure 4).

HPV-mediated OPSCC is fairly responsive to chemoradiotherapy and has a better prognosis than HPV-unrelated OPSCC [32]. Approximately 30% of OPSCC patients are HPV positive in Taiwan. In the Gene Expression Omnibus database (GEO) [33], in the GDS1667/219271_at profile, *GALNT14* mRNA expression was higher in HPV-positive than in HPV-negative head and neck cancer cases (Figure S3a,b). In the GDS3126/219271_at profile, patients with high *GALNT14* expression also showed radiosensitivity compared to patients with low *GALNT14* expression (Figure S3c,d). Upregulation of *GALNT14* mRNA in tumors was higher in the dead patients in the radiotherapy and overall survival die groups (Table 3) and reduced the radiotherapy- and overall-survival times in OPSCC (Figure 2g). The decreased survival rate after treatment may be related to patient age, tumor malignancy, tumor resistance, and the side effects of treatment. Tenofovir is a major negative regulator of GALNT14 substrates and an unfavorable anti-hepatitis B drug in patients with hepatocellular carcinoma receiving sorafenib [34]. Therefore, high expression of *GALNT14* may also affect chemotherapy outcome (Figure 5d). Currently, there are no studies on *GALNT* expression in OPSCC. Whether HPV infection affects *GALNT14* mRNA expression in OPSCC patients requires further exploration. The detailed medical records, risk factors, and chemoradiotherapy plan for each HNSCC patient require further investigation.

*GALNT14*-rs9679162, located in intron 3 of the *GALNT14* gene, has no effect on mRNA and amino acid sequences. However, introns have direct and indirect roles in regulating themselves or other genes. The direct functions include alternative splicing, enhanced gene expression, control of mRNA transport, chromatin assembly, and nonsense-mediated decay. The indirect role function includes natural selection, a source of new genes, and non-coding functional RNA genes [35]. We did not find a relationship between *GALNT14*-rs9679162 polymorphism or mRNA expression and the risk factors and the treatment-survival curves in clinical data (Figure 3, Figure 4 and Figure 5). In the in vitro model, the *GALNT14* mRNA was downregulated in four out of seven (57%) oral cell lines. However, *GALNT14* mRNA and protein expression downregulated in cell lines with the TT genotype and upregulated in cell lines with non-TT genotype, upon treatment with arecoline (Figure 4h–n). There have been no in vitro studies focusing on the different *GALNT14*-rs9679162 genotypes; therefore, the effect of different *GALNT14*-rs9679162 genotypes on cell phenotype, genomics, and proteomics remains unclear. *GALNT14* has two alternative splicing forms, the transcript contains exons 4 but not exons 2 and 3, and the transcript contains exons 2 and 3 but not exon 4 [36]. The direct and indirect roles of arecoline in regulating GALNT14 protein expression and *GALNT14*-rs9679162 genotype selection in betel nut users with OSCC require further study.

mTPF-based chemotherapy and radiotherapy are often combined to treat OSCC. Sequential treatment with docetaxel and 5-Fu is commonly used to treat human oral cancer [37]. Compared with cisplatin and 5-Fu combination treatment, induction chemotherapy with the addition of docetaxel significantly enhanced progression-free overall survival in patients with unresectable HNSCC [38]. Some studies have shown that high GALNT14 expression induces apoptosis and chemosensitivity. GALNT14 promotes the O-glycosylation of death receptors 4/5 (DR4/5) [39] and mediates tumor necrosis factor-related apoptosis-inducing ligand (TRAIL)-induced apoptosis in pancreatic carcinoma, non–small cell lung carcinoma, and melanoma cells [40]. GALNT14 protein expression was significantly higher in cell lines sensitive to dulanermin and drozitumab compared to that in resistant non–small cell lung cancer (NSCLC) cell lines [41]. However, some studies have shown that high GALNT14 expression induces chemoresistance in cancer cells. Overexpression of glycosylated P-glycoprotein (P-gp) in drug-treated cancer cells is one of the major causes for the failure of cancer chemotherapy. GALNT14 is associated with higher P-gp levels in adriamycin-resistant human breast cancer tissues [5]. To clarify whether *GALNT14* GG genotype or mRNA expression affects chemotherapy survival, cells were only administered a single drug for chemoresistance analysis. OSCC cell lines with the TT genotype were docetaxel resistant, and OSCC cell lines with high *GALNT14* mRNA expression and the TG genotype showed 5-Fu resistance (Figure 5). If these preliminary results were not coincidental, there may be some correlation between *GALNT14* genotype and *GALNT14* expression. 5-FU resistance is controlled by three major metabolic enzymes: thymidylate synthase (TS), dihydropyrimidine dehydrogenase (DPD), and thymidine phosphorylase (TP) [42,43]. Drug efflux mediated by the transporter proteins, such as multidrug resistance 1 (MDR1) and MDR protein 5 (MRP5), plays a critical role in docetaxel resistance [44,45]. Based on the results of previous studies, it is speculated that *GALNT14*-rs9679162 TT genotype and upregulation *GALNT14* enhanced the chemoresistance mechanisms against docetaxel and 5-Fu, respectively. Furthermore, *GALNT14* mutations have been associated with neuroblastoma predisposition [46]. *GALNTL14* is more commonly mutated in the non-complete response group to neoadjuvant chemoradiotherapy in locally advanced rectal cancer [47]. Whether the mutation rate in the three *GALNT14*-rs9679162 genotypes is also an important issue for future studies.

In this study, the frequency of *GALNT14*-rs9679162 genotypes and the expression of *GALNT14* were analyzed using HNSCC tissues, HNSCC cell lines, and the public HNSCC data platforms. The frequency of *GALNT14*-rs9679162 genotypes and *GALNT14* expression at different HNSCC sites were different. The *GALNT14*-rs9679162 non-TT genotype was associated with survival, and the *GALNT14*-rs9679162 allele was associated with alcohol consumption, betel nut consumption, and cigarette smoking. *GALNT14* was upregulated in OPSCC but downregulated in OSCC and LSCC, which may be related to different carcinogenic risk factors. HPV infection or alcohol consumption in HNSCC may upregulate the expression of *GALNT14*, while betel nut chewing may downregulate the expression of *GALNT14* in individuals with the TT genotype but upregulate the expression of *GALNT14* in individuals with the non-TT genotype. *GALNT14*-rs9679162 non-TT genotypes and high *GALNT14* expression may enhance chemoresistance in HNSCC via different mechanisms. *GALNT14*-rs9679162 non-TT genotypes and *GALNT14* expression can be used as indicators of prognosis and survival in HNSCC patients. In the future, the sample size from each HNSCC site should be increased to clarify the association of *GALNT14*-rs9679162 non-TT genotypes with *GALNT14* expression and response to chemoradiotherapy.

Neoadjuvant chemotherapy is administered preoperatively to reduce the tumor volume and to facilitate the main treatment, such as surgery or radiotherapy. The vascular bed surrounding the tumor provides efficient drug delivery. However, neoadjuvant chemotherapy also delays the main therapy, and the physician must ensure that the patient has good response and that the tumor is not progressing during neoadjuvant chemo-therapy. Therefore, predicting the patient’s response to neoadjuvant chemotherapy can shorten the treatment time, reduce the side effects, and avoid the occurrence of drug resistance. This study showed that *GALNT14*-rs9679162 genotype and *GALNT14* mRNA expression are associated with post-treatment survival in head and neck cancer, and can be used as indicators to predict the response to neoadjuvant chemotherapy.

## Figures and Tables

**Table 1:** Comparison of clinical parameters between *GALNT14*-rs9679162 TT and non-TT genotypes in HNSCC.

	HNSCC					OSCC					OPSCC					LSCC				
	TT	Non-TT	*p*	OR	95% CI	TT	Non-TT	*p*	OR	95% CI	TT	Non-TT	*p*	OR	95% CI	TT	Non-TT	*p*	OR	95% CI
Age																				
<57	30	69	0.8775	1.064	0.5843–1.909	21	40	0.5503	1.295	0.5706–2.827	5	13	>0.9999	1.231	0.2892–5.249	3	15	0.6928	0.56	0.1312–2.932
≥57	29	71				15	37				5	16				5	14			
Alcohol																				
no	19	32	0.213	1.603	0.8075–3.206	15	16	0.0251	2.723	1.129–6.646	0	6	0.3084	0	0.000–1.536	1	10	0.3909	0.2714	0.02214–2.171
yes	40	108				21	61				10	23				7	19			
Betel nut																				
no	16	35	0.859	1.116	0.5715–2.265	10	7	0.0209	3.846	1.386–10.14	0	8	0.0862	0.1204	0.000–1.361	3	10	>0.9999	1.14	0.2588–6.238
yes	43	105				26	70				10	21				5	19			
Cigarette																				
no	12	17	0.1854	1.847	0.7918–4.034	9	3	0.0016	8.222	2.326–29.24	0	2	>0.9999	0	0.000–6.348	1	8	0.6487	0.375	0.02995–3.200
yes	47	123				27	74				10	27				7	21			
Differentiation																				
no record	5	9				4	3				0	2				0	3			
well	6	10				6	9				0	1				0	0			
moderate	45	111	0.6152	1.405	0.3837–4.913	25	61	>0.9999	1.771	0.2719–22.30	9	21	>0.9999	2.045	0.2907–26.49	7	25	0.4207	0.28	0.01428–6.031
poor	3	10				1	4				1	5				1	1			
TNM, T																				
no record	8	14				5	6				0	3				2	4			
T0 + T1 + T2	26	66	0.8699	0.9455	0.4957–1.803	13	31	>0.9999	0.9319	0.4040–2.164	4	18	0.1401	0.2963	0.07972–1.258	5	15	0.3826	3.333	0.4626–42.71
T3 + T4	25	60				18	40				6	8				1	10			
TNM, N																				
no record	7	17				5	9				0	4				1	3			
N = 0	29	60	0.4134	1.324	0.6857–2.470	20	41	0.8243	1.197	0.4976–2.873	5	8	0.4437	2.125	0.4530–10.35	1	9	0.3968	0.3148	0.02497–2.911
N > 0	23	63				11	27				5	17				6	17			
Stage																				
no record	6	4				3	2				0	0				2	2			
stage BBB	3	11				3	5				0	3				0	2			
stage I + II + III	23	56	>0.9999	1.05	0.5568–2.067	15	31	0.6644	1.258	0.5238–3.033	3	12	0.4682	0.5	0.1220–2.082	2	12	0.6638	0.5417	0.09199–2.914
stage IV	27	69				15	39				7	14				4	13			
Radiotherapy- survival status																				
live	26	47	0.0462	2.489	1.078–5.715	15	33	>0.9999	1.136	0.3868–3.722	5	6	0.084	5.833	0.9464–33.23	5	6	0.084	5.833	0.9464–33.23
dead	10	45				6	15				2	14				2	14			
Chemotherapy-survival status																				
live	28	54	0.1279	1.901	0.8957–4.269	15	36	>0.9999	1.111	0.3883–3.572	6	10	0.4646	1.95	0.4592–7.322	5	6	0.0946	5.417	0.8872–31.04
dead	12	44				6	16				4	13				2	13			
Survival status																				
live	41	77	0.0603	1.864	1.002–3.566	27	54	0.659	1.278	0.5305–2.989	6	11	0.2819	2.455	0.6224–8.895	6	10	0.0554	5.7	1.020–30.23
dead	18	63				9	23				4	18				2	19			
Site						0	0													
tongue						14	18	0.0881	2.086	0.8633–4.912										
cheek						7	27													
other sites						15	32													
Type																				
OSCC	36	77	0.5311	1.281	0.6778–2.401															
OPSCC	10	29																		
LSCC	8	29																		
others	5	5																		

**Table 2:** Alleotypes of *GALNT14*-rs9679162 in HNSCC.

	HNSCC					OSCC					OPSCC					LSCC				
	T	G	*p*	OR	95% CI	T	G	*p*	OR	95% CI	T	G	*p*	OR	95% CI	T	G	*p*	OR	95% CI
Age																				
<57	102	96	>0.9999	1.001	0.6798–1.472	67	55	0.5938	1.172	0.6852–2.012	19	17	0.6499	1.353	0.5334–3.143	15	21	0.4941	0.7143	0.2907–1.703
≥57	103	97				53	51				19	23				19	19			
Alcohol																				
no	56	46	0.4908	1.201	0.7667–1.903	38	24	0.138	1.583	0.8816–2.825	3	9	0.1157	0.2952	0.08151–1.062	8	14	0.3176	0.5714	0.1947–1.630
yes	149	147				82	82				35	31				26	26			
Betel nut																				
no	49	53	0.424	0.83	0.5267–1.301	24	10	0.0392	2.4	1.066–5.095	5	11	0.1624	0.3994	0.1402–1.247	12	14	>0.9999	1.013	0.3776–2.643
yes	156	140				96	96				33	29				22	26			
Cigarette																				
no	34	24	0.2582	1.392	0.7926–2.475	19	5	0.0085	3.8	1.401–9.565	2	2	>0.9999	1.056	0.1588–7.007	8	10	>0.9999	0.9231	0.2953–2.616
yes	171	169				101	101				36	38				26	30			
Differentiation																				
no record	14	14				10	4				1	3				0	6			
well	18	16	0.7157	0.8389	0.4009–1.673	18	12	0.4309	1.467	0.6698–3.267	0	2				0	0			
moderate	159	117				87	85				30	30	0.7544	0.7143	0.2232–2.450	32	32	>0.9999	1.000	0.1496–6.683
poor	14	12				5	5				7	5				2	2			
TNM, T																				
no record	25	19				14	8				3	3								
T0 + T1 + T2	90	94	0.4584	0.8511	0.5647–1.279	44	44	0.6723	0.871	0.4953–1.527	20	24	0.6295	0.7222	0.2736–1.844	18	22	0.7929	0.8182	0.2986–2.227
T3 + T4	90	80				62	54				15	13				11	11			
TNM, N																				
no record	25	23				15	13				4	4								
N = 0	95	83	0.5211	1.172	0.7761–1.773	63	59	0.6620	0.8644	0.4902–1.508	14	12	0.6218	1.400	0.5235–3.904	10	10	0.7931	1.190	0.4250–3.346
N > 0	85	87				42	34				20	24				21	25			
Stage																				
no record	14	8				8	2													
stage BBB	14	14				9	7				3	3								
stage I + II + III	79	79	0.9145	0.9592	0.6325–1.454	44	48	0.3947	0.7613	0.4293–1.338	14	16	0.8149	0.8750	0.3434–2.187	14	14	0.7987	1.267	0.4417–3.266
stage IV	98	94				59	49				21	21				15	19			
Radiotherapy-survival status																				
live	84	62	0.0114	1.957	1.167–3.235	53	43	0.5838	1.233	0.6167–2.468	14	8	0.0520	3.341	1.100–9.802	15	7	0.0258	4.091	1.316–13.06
dead	45	65				21	21				11	21				11	21			
Chemotherapy- survival status																				
live	95	69	0.0497	1.647	1.015–2.703	57	45	0.3734	1.387	0.6737–2.901	19	13	0.4697	1.462	0.5498–4.080	15	7	0.0483	3.701	1.168–11.99
dead	51	61				21	23				17	17				11	19			
Survival status																				
live	133	103	0.0246	1.614	1.084–2.423	91	71	0.1828	1.547	0.8754–2.782	19	15	0.3612	1.667	0.6506–3.983	19	13	0.06	2.631	1.067–7.079
dead	72	90				29	35				19	25				15	27			
Site																				
tongue						39	25	0.1428	1.560	0.8790–2.741										
cheek						31	37													
others						50	44													
Type																				
OSCC	120	106	0.4801	1.159	0.7849–1.713															
OPSCC	38	40																		
LSCC	34	40																		
others	13	7																		

**Table 3:** Association between *GALNT14* expression and clinical parameters in HNSCC and subtype cancers.

	HNSCC					OSCC					OPSCC					LSCC				
	N > T	N < T	*p*	OR	95% CI	N > T	N < T	*p*	OR	95% CI	N >T	N < T	*p*	OR	95% CI	N > T	N < T	*p*	OR	95% CI
Age																				
<57	20	12	0.6265	1.333	0.5037–3.720	9	6	0.6828	0.5	0.1126–2.718	5	4	0.1597	5.625	0.8801–34.38	7	2	0.6424	2	0.2900–12.77
≥57	20	16				9	3				2	9				7	4			
Alcohol																				
no	16	7	0.2977	2.000	0.6660–5.350	9	4	>0.9999	1.25	0.2186–5.171	3	1	0.1011	6.481	0.7215–58.22	4	2	0.9999	0.8	0.09034–5.585
yes	24	21				9	5				4	12				10	4			
Betel nut																				
no	15	10	>0.9999	1.080	0.3830–2.821	8	4	>0.9999	1	0.1999–4.151	2	2	0.5868	2.2	0.2709–16.53	5	4	0.3359	0.2778	0.04416–1.968
yes	25	18				10	5				5	11				9	2			
Cigarette																				
no	8	6	>0.9999	0.9167	0.3059–3.281	4	4	0.3748	0.3571	0.06951–1.723	2	0	0.1105	infinity	0.9268–Infinity	2	2	0.5492	0.3333	0.04393–2.830
yes	32	22				14	5				5	13				12	4			
Differentiation																				
no record	2	2				1	2				0	0				1	0			
well	6	1	0.2254	4.688	0.6564–55.69	5	1	0.6287	2.5	0.3013–33.80	1	0	0.35	infinity	0.2063–Infinity	0	0	>0.9999	0.000	to Infinity
moderate	29	24				12	6				3	12				13	6			
poor	3	1				0	0				3	1				0	0			
TNM, T																				
no record	5	8				3	3				0	1				2	4			
T0 + T1 + T2	21	14	0.5653	0.6429	0.1818–2.087	7	2	0.6594	1.75	0.2620–11.11	3	10	0.1287	0.15	0.02296–1.257	11	2	>0.9999	0.000	0.000–54.00
T3 + T4	14	6				8	4				4	2				1	0			
TNM, N																				
no record	9	5				4	3				1	1				4	1			
N = 0	18	6	0.0275	3.923	1.246–12.22	7	2	0.6424	2	0.2900–12.77	5	4	0.1312	10	1.000–129.5	5	0	0.1009	infinity	0.6674–Infinity
N > 0	13	17				7	4				1	8				5	5			
Stage																				
no record	3	6				2	3				0	0				1	3			
stage BBB	2	2				1	0				0	1				1	1			
stage I + II + III	18	5	0.0879	3.176	0.9592–9.664	7	1	0.3359	4.375	0.5157–57.88	4	4	0.3765	2.667	0.4111–14.26	7	0	0.4615	infinity	0.4873–Infinity
stage IV	17	15				8	5				3	8				5	2			
Radiotherapy-survival status																				
live	8	5	0.5263	1.6	0.4213–5.814	3	4	0.1414	0.1667	0.02521–1.429	3	1	0.014	infinity	2.759–Infinity	2	0	0.4909	infinity	0.2670–Infinity
dead	15	15				9	2				0	9				5	4			
Chemotherapy-survival status																				
live	9	6	>0.9999	1.196	0.3495–4.091	2	4	0.1070	0.1	0.01412–1.017	5	2	0.0584	10	1.117–69.44	2	0	0.4643	infinity	0.2840–Infinity
dead	16	13				10	2				2	8				3	3			
Survival status																				
Live	18	9	0.3234	1.727	0.6249–4.711	7	5	0.4479	0.5091	0.1194–2.930	5	2	0.0223	13.75	1.646–92.10	6	2	>0.9999	1.5	0.2120–9.753
Dead	22	19				11	4				2	11				8	4			
Site																				
tongue	--	--	--	--		3	1	>0.9999	1.6	0.2043–23.10										
cheek	--	--	--	--		4	4													
others	--	--	--	--		11	4													
Type																				
OSCC	18	9	0.0419	3.714	1.048–13.56															
OPSCC	7	13																		
LSCC	14	6	0.0562	4.333	1.217–15.12															
others	1	0																		

## Data Availability

The data presented in this study are available on request from the corresponding author.

## Supplementary Materials

The following supporting information can be downloaded at: https://www.mdpi.com/article/10.3390/cancers14174217/s1, Supplementary Figure S1. All Western blot figures (uncropped blots), include densitometry readings/intensity ratio of each band. Figure S2. *GALNT14* expression level was associated with alcohol consumption, tumor stage, and survival time in head and neck cancer. Figure S3. The relation between *GALNT14* expression and HPV positivity, or radiosensitivity in head and neck cancer, Table S1. Clinical characteristics of the subjects in *GALNT14*-rs9679162 polymorphism analysis, Table S2. Clinical characteristics of the subjects in *GALNT14* mRNA expression analysis.
