# Supplementary material for: Association of rs9679162 Genetic Polymorphism and Aberrant Expression of Polypeptide N-Acetylgalactosaminyltransferase 14 (GALNT14) in Head and Neck Cancer
Source: Cancers (Basel). 2022 Aug 30;14(17):4217. doi: 10.3390/cancers14174217 (PMC9454803; doi:10.3390/cancers14174217)
Supplement: Supplementary file 1 [file cancers-14-04217-s001.zip › cancers-1849351-Supplementary/cancers-1849351-Figures S1-S3.pdf]

Supplementary Figures

Supplementary Figure S1.

Figure 4g. GALNT14 protein expression in 7 OSCC cell lines.

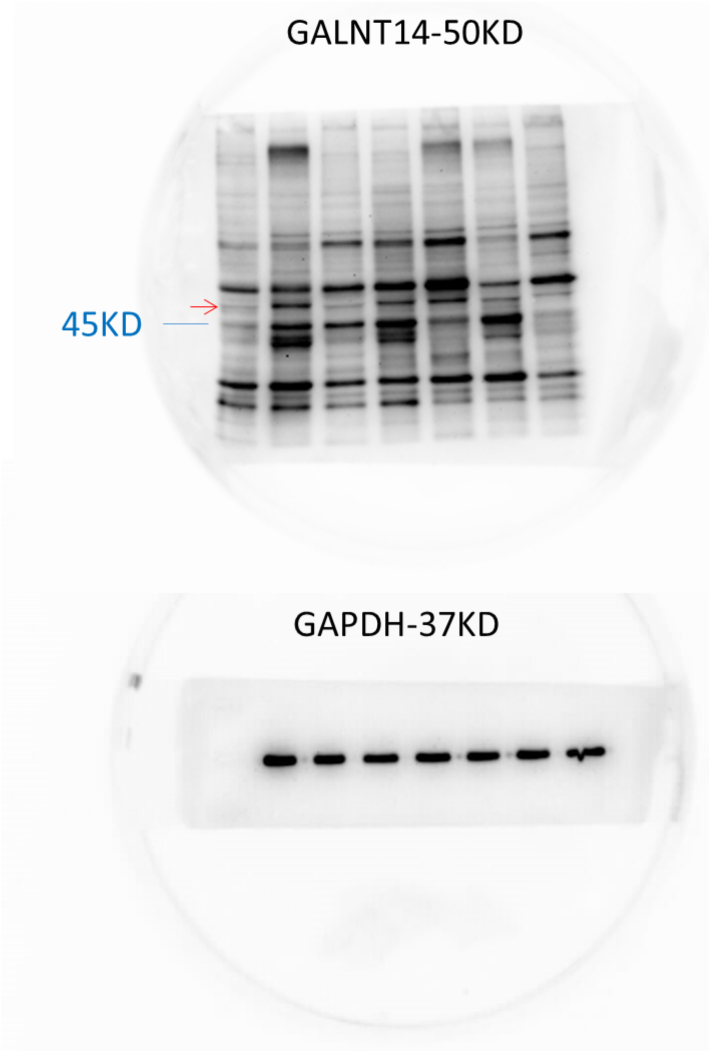

	SG	SAS	HSC3	OECM1	A253	FADU	SCC9
GALNT 14	9836.276	23054.882	15042.933	21822.569	24398.225	16612.861	8741.376
GAPDH	20032.811	14529.033	13521.447	15131.933	13794.69	13845.276	15345.811
ratio	0.491008276	1.586814621	1.112523904	1.442153425	1.768667871	1.199893812	0.569626200
comparison	1.00	3.23	2.27	2.94	3.60	2.44	1.16

Figure 4h. GALNT14 protein expression after treatment with vehicle, 12.5 or 100 µg/mL arecoline for 24 h in SG.

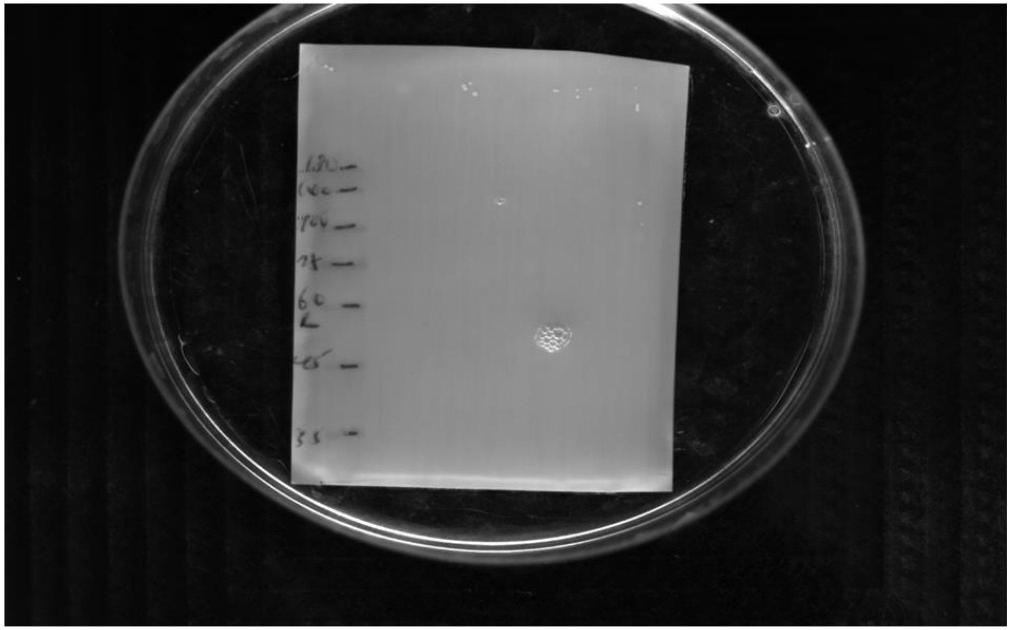

GALNT14-50KD

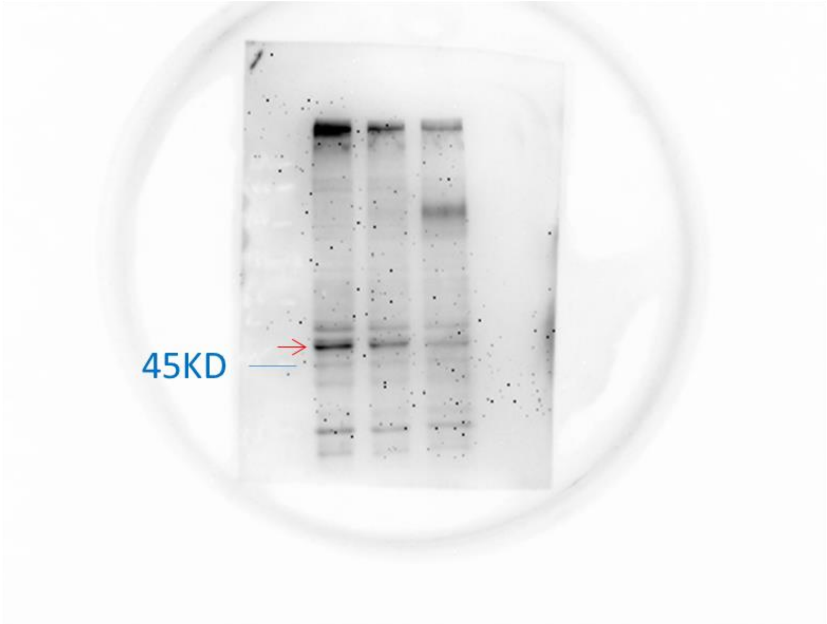

GAPDH-37KD

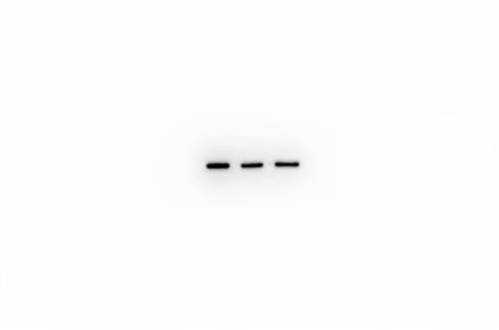

SG			
	CON	ARE 12.5	ARE 100
GALNT 14	43987.936	28694.744	19507.459
GAPDH	47203.066	37068.095	36878.288
ratio	0.931887263	0.774108947	0.528968671
comparison	1.00	0.83	0.57

Figure 4i. GALNT14 protein expression after treatment with vehicle, 12.5 or 100 µg/mL arecoline for 24 h in SAS.

GALNT14-50KD

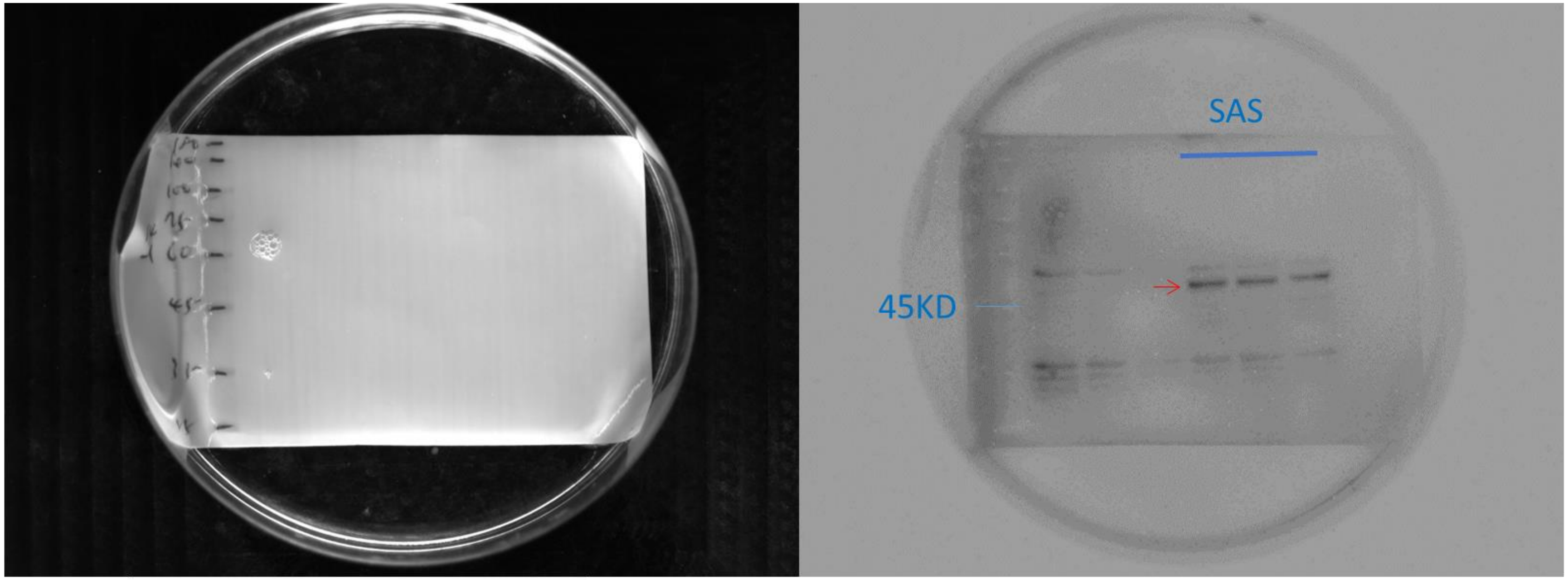

GAPDH-37KD

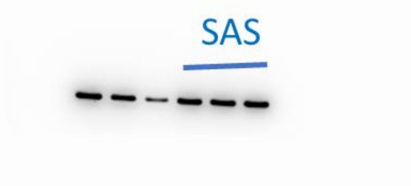

SAS			
	CON	ARE 12.5	ARE 100
GALNT 14	43444.673	34944.593	25722.986
GAPDH	26328.69	26103.74	23258.69
ratio	1.650088668	1.338681469	1.105951625
comparison	1.00	0.81	0.67

Figure 4j. GALNT14 protein expression after treatment with vehicle, 12.5 or 100 μg/mL arecoline for 24 h in HSC3.

GALNT14-50KD

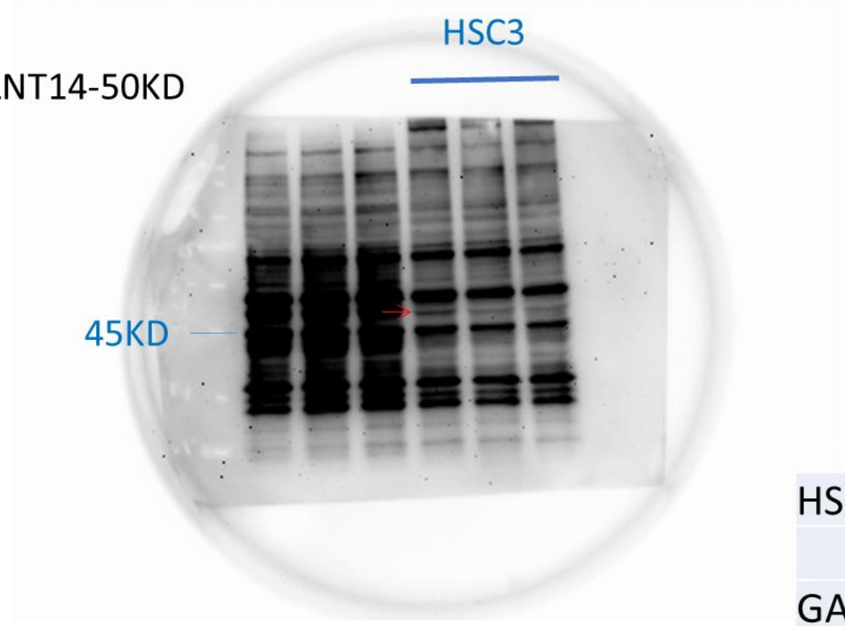

GAPDH-37KD

HSC3

HSC3			
	CON	ARE 12.5	ARE 100
GALNT 14	51283.045	34344.238	30025.459
GAPDH	44606.087	39955.329	39128.865
ratio	1.149687149	0.859565892	0.767348069
comparison	1.00	0.75	0.67

Figure 4k. GALNT14 protein expression after treatment with vehicle, 12.5 or 100 µg/mL arecoline for 24 h in OECM-1.

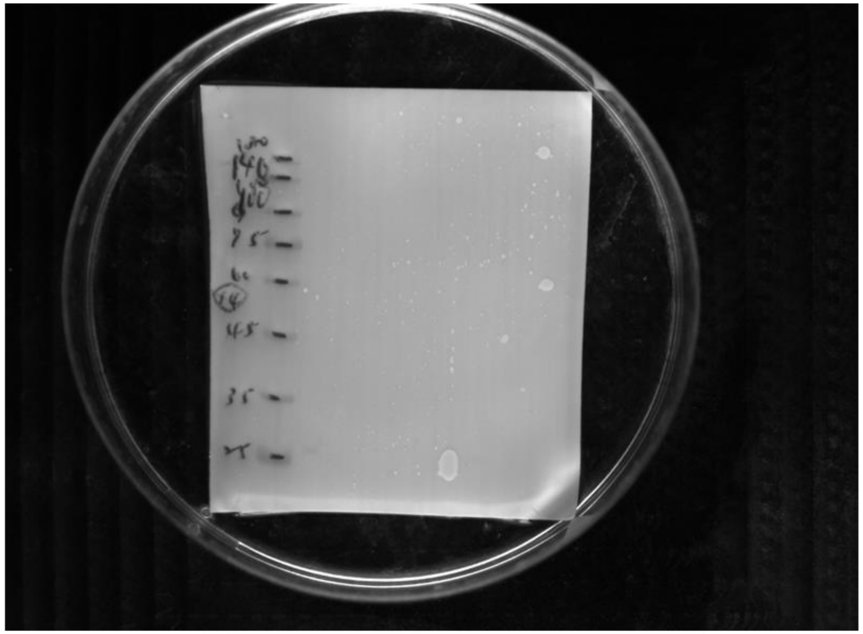

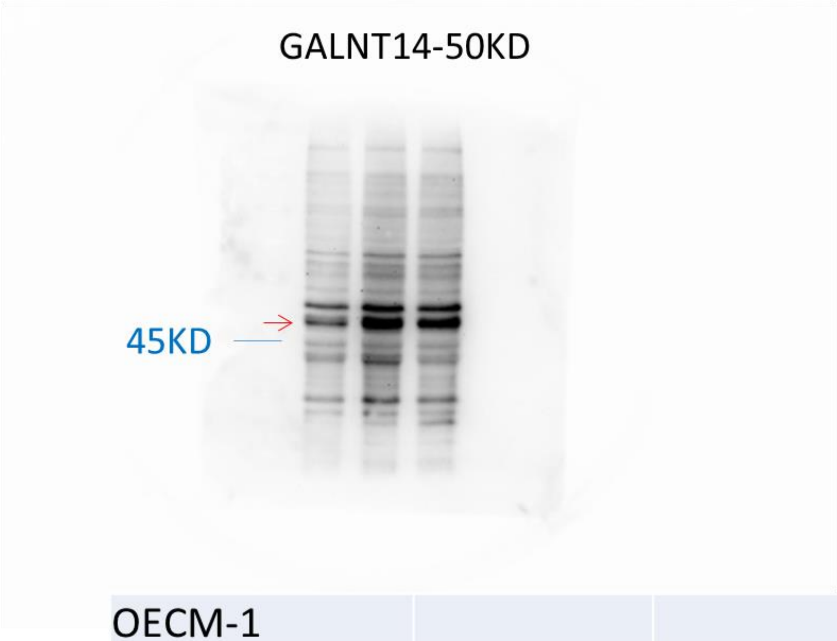

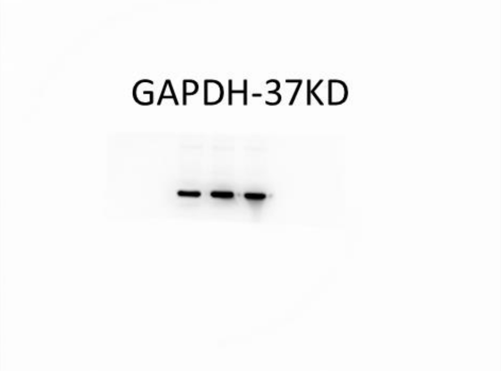

OECM-1			
	CON	ARE 12.5	ARE 100
GALNT 14	25937.116	53566.217	47624.338
GAPDH	31793.388	43786.238	38974.409
ratio	0.815802204	1.223357371	1.221938683
comparison	1.00	1.50	1.50

Figure 4l. GALNT14 protein expression after treatment with vehicle, 12.5 or 100 µg/mL arecoline for 24 h in A253.

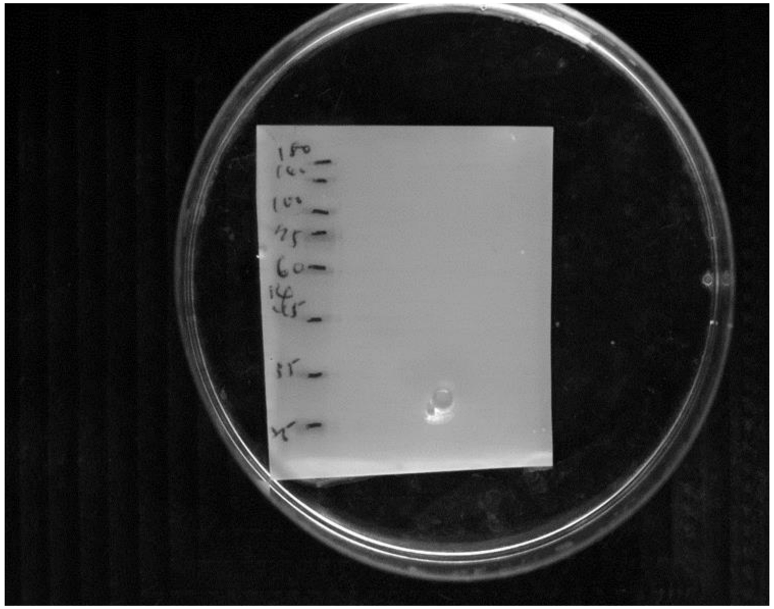

GALNT14-50KD

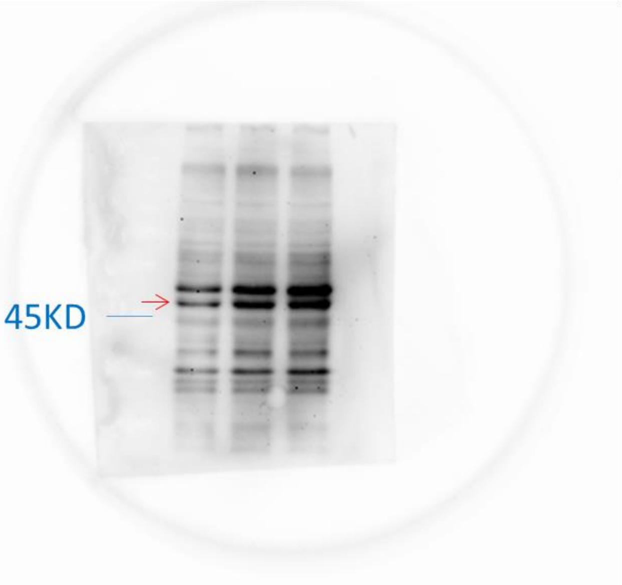

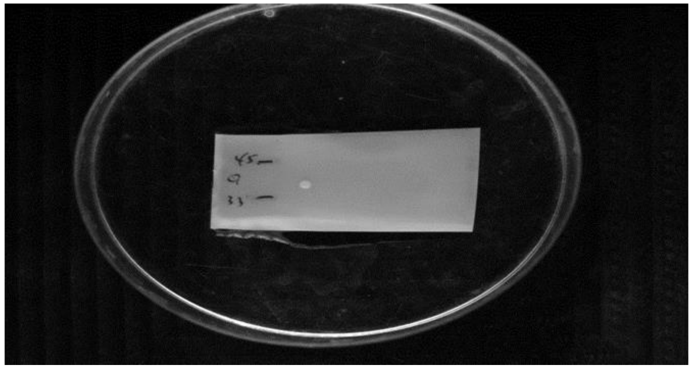

GAPDH-37KD

A253			
	CON	ARE 12.5	ARE 100
GALNT 14	18777.794	43347.702	51888.945
GAPDH	40530.338	44432.581	42928.773
ratio	0.463302181	0.975583705	1.208721829
comparison	1.00	2.11	2.61

Figure 4m. GALNT14 protein expression after treatment with vehicle, 12.5 or 100 µg/mL arecoline for 24 h in FaDu.

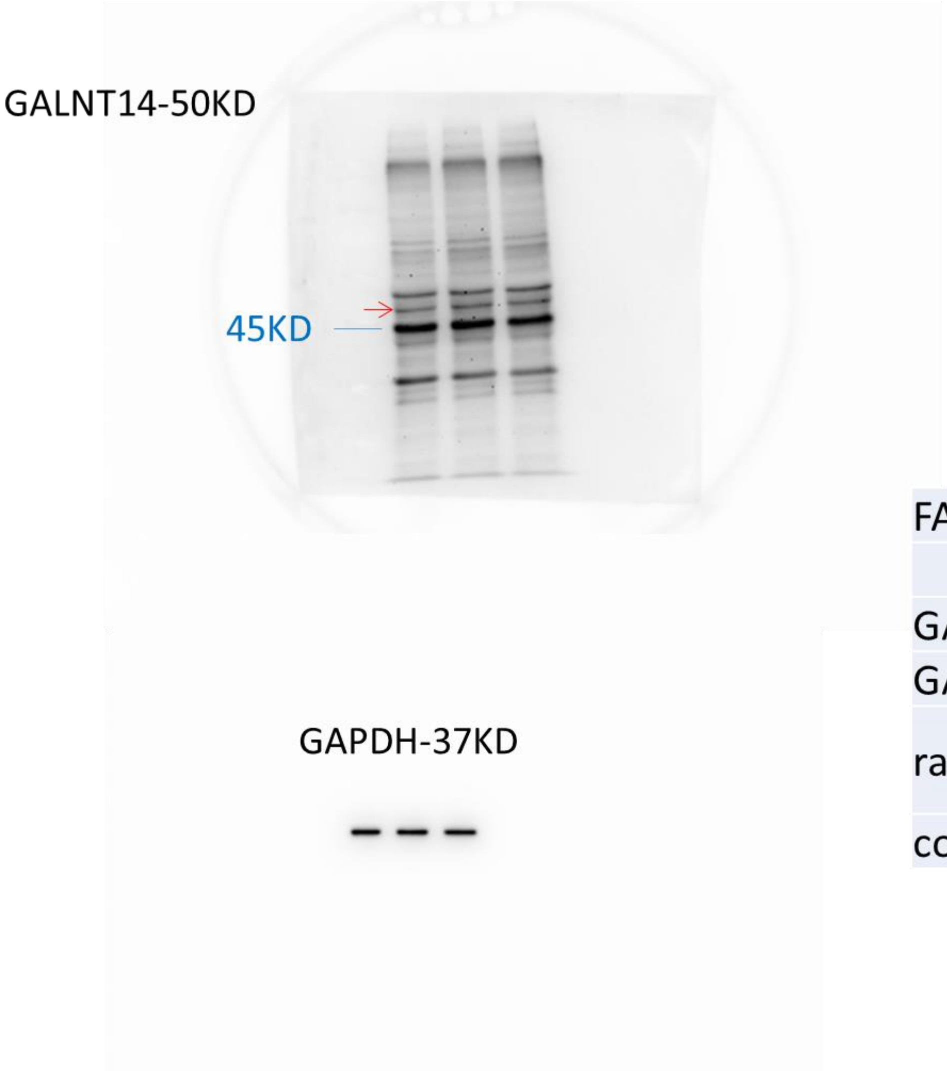

FADU			
	CON	ARE 12.5	ARE 100
GALNT 14	36059.681	46394.451	43711.167
GAPDH	39124.459	43089.409	40708.167
ratio	0.921665933	1.076701957	1.073768981
comparison	1.00	1.17	1.17

Figure 4n. GALNT14 protein expression after treatment with vehicle, 12.5 or 100 µg/mL arecoline for 24 h in SCC9.

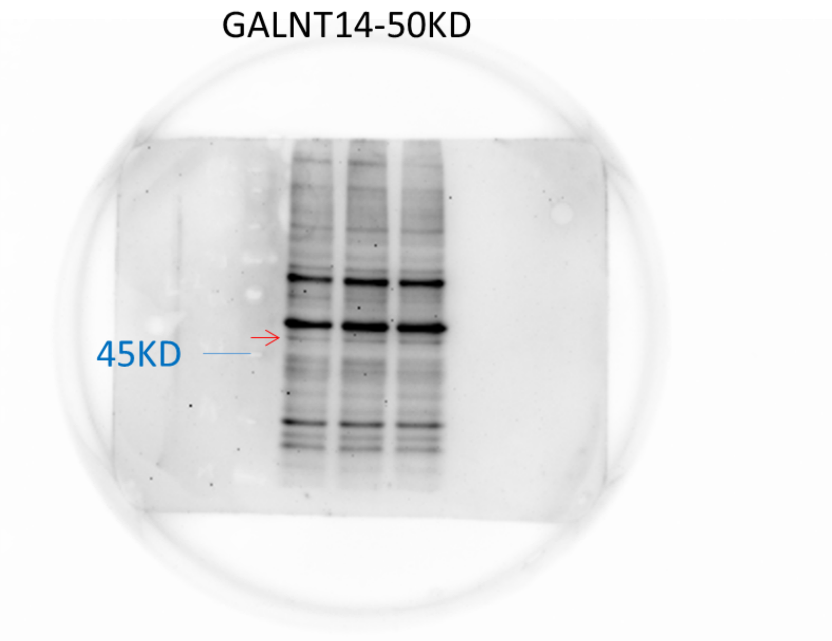

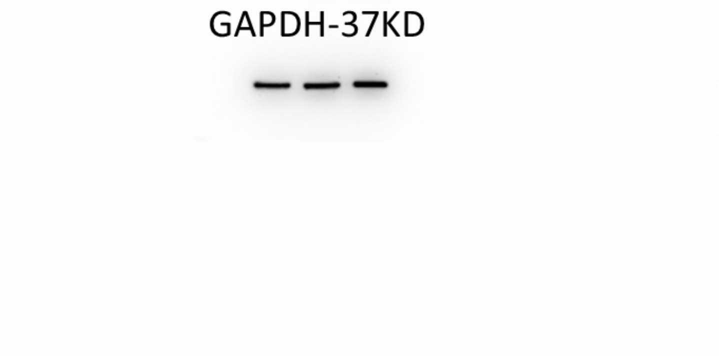

SCC9			
	CON	ARE 12.5	ARE 100
GALNT 14	24887.108	33044.229	43366.037
GAPDH	38861.409	46550.095	46304.844
ratio	0.640406734	0.709863836	0.936533487
comparison	1.00	1.11	1.46

Figure S1. All Western blot figures (uncropped blots) in Figure 4g-n, include densitometry readings/intensity ratio of each band.

Supplementary Figure S2.

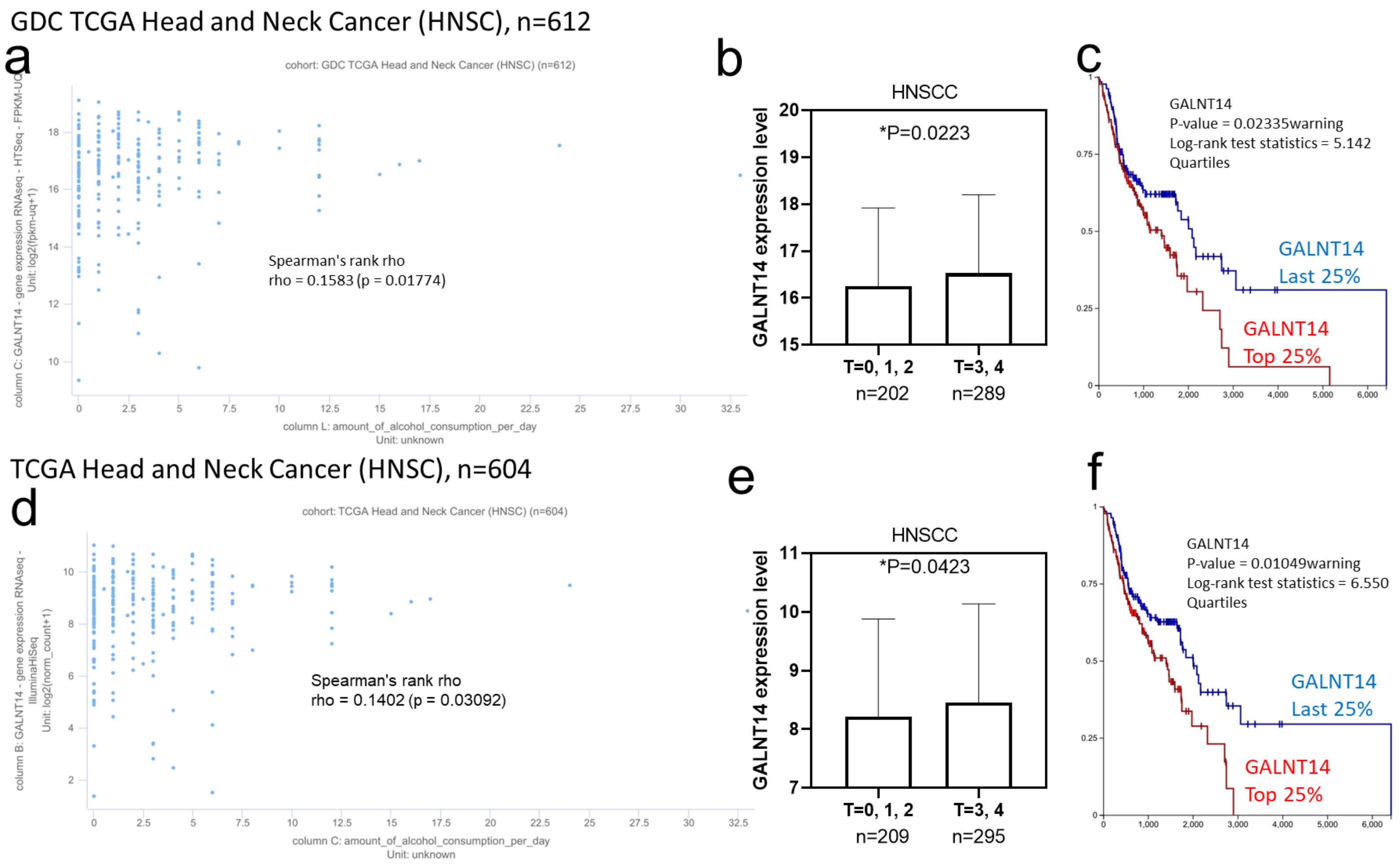

Figure S2. *GALNT14* expression level was associated with alcohol consumption, tumor stage, and survival time in head and neck cancer. (a, b, c) GDC TCGA Head and Neck Cancer profile. (d, e, f) TCGA Head and Neck Cancer profile. (a), (d) The relation between *GALNT14* expression level and alcohol consumption analyzed using Spearman's rank correlation test. (c), (e) *GALNT14* expression level in tumor early stage (0, 1, and 2) and late stage (3, and 4), analyzed using Mann-Whitney test. (c), (f) The survival time of *GALNT14* expression level last 25% and top 25% groups, Analyzed using Log-rank test. * $p < 0.05$.

Supplementary Figure S3.

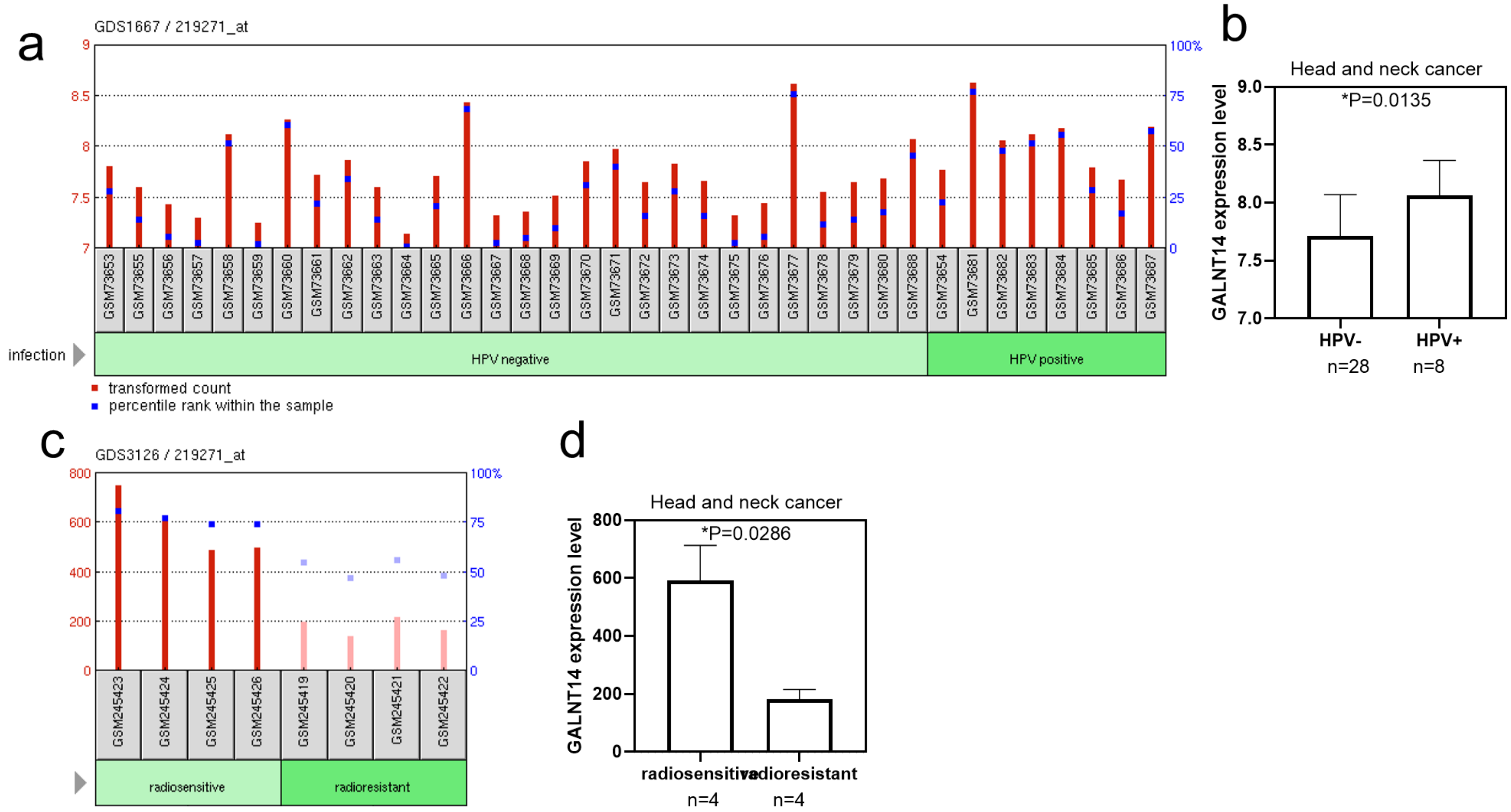

Figure S3. The relation between *GALNT14* expression and HPV positivity, or radiosensitivity in head and neck cancer. The data generated in this study are publicly available in GEO at GDS1667 / 219271_at and GDS3126 / 219271_at profiles. (a) The *GALNT14* expression level of each case in the GEO profile. HPV-, n=28; HPV+, n=8. (b) *GALNT14* expression level (mean ± SD) in HPV- and HPV+ groups. (c) The *GALNT14* expression level of each case in the GEO profile. Radiosensitive, n=4; Radioresistant, n=4. (d) *GALNT14* expression level (mean ± SD) in radiosensitive and radioresistant groups. (b), (d), analyzed using Mann-Whitney test. * $p < 0.05$.
