# Supplementary material for: Association of rs9679162 Genetic Polymorphism and Aberrant Expression of Polypeptide N-Acetylgalactosaminyltransferase 14 (GALNT14) in Head and Neck Cancer
Source: Cancers (Basel). 2022 Aug 30;14(17):4217. doi: 10.3390/cancers14174217 (PMC9454803; doi:10.3390/cancers14174217)
Supplement: Supplementary file 1 [file cancers-14-04217-s001.zip › cancers-1849351-Supplementary/cancers-1849351-Table S1 and Table S2.pdf]

Supplementary Tables

Table S1. Clinical characteristics of the subjects in *GALNT14*-rs9679162 polymorphism analysis.

	HNSCC (mean of age=57.33±10.50)			
	OSCC	OPSCC	LSCC	others
**Mean of age**	56.68 ± 10.67	57.62 ± 7.358	58.76 ±10.90	58.30 ±16.98
Gender				
male	106 (93.8%)	36 (92.3%)	37 (100.0%)	9 (90.0%)
female	7 (6.2%)	3 (7.7%)	0 (0.0%)	1 (10.0%)
**Alcohol**				
no	30 (26.5%)	6 (15.4%)	11 (29.7%)	4 (40.0%)
yes	83 (73.5%)	33 (84.6%)	26 (70.3%)	6 (60.0%)
**Betel nut**				
no	23 (20.4%)	8 (20.5%)	13 (35.1%)	7 (70.0%)
yes	90 (79.6%)	31 (79.5%)	24 (64.9%)	3 (30.0%)
**Cigarette**				
no	15 (13.3%)	2 (5.1%)	9 (24.3%)	3 (30.0%)
yes	98 (86.7%)	37 (94.9%)	28 (75.7%)	7 (70.0%)
**Differentiation**				
no record	7 (6.2%)	2 (5.1%)	3 (8.1%)	2 (20.0%)
well	15 (13.3%)	1 (2.6%)	0 (0.0%)	0 (0.0%)
moderate	86 (76.1%)	30 (76.9%)	32 (86.5%)	8 (80.0%)
poor	5 (4.4%)	6 (15.4%)	2 (5.4%)	0 (0.0%)
**TNM**				
no record	11 (9.7%)	3 (7.7%)	6 (16.2%)	2 (20.0%)
T0	2 (1.8%)	1 (2.6%)	0 (0.0%)	0 (0.0%)
T1	20 (17.7%)	3 (7.7%)	11 (29.7%)	6 (60.0%)
T2	22 (19.5%)	18 (46.2%)	9 (24.3%)	0 (0.0%)
T3	19 (16.8%)	6 (15.4%)	7 (18.9%)	1 (10.0%)
T4	39 (34.5%)	8 20.5%	4 (10.8%)	1 (10.0%)
**TNM**				
no record	14 (12.4%)	4 (10.3%)	4 (10.8%)	2 (20.0%)
N=0	61 (54.0%)	13 (33.3%)	10 (27.0%)	5 (50.0%)
N=1	9 (8.0%)	6 (15.4%)	4 (10.8%)	0 (0.0%)
N=2	18 (16.0%)	14 (35.9%)	16 (43.2%)	3 (30.0%)
N=3	11 (9.7%)	2 (5.1%)	3 (8.1%)	0 (0.0%)
**Stage**				
no record	5 (4.4%)	0 (0.0%)	4 (10.8%)	1 (10.0%)
stage BBB	8 (7.1%)	3 (7.7%)	2 (5.4%)	1 (10.0%)
stage I	16 (14.2%)	5 (12.8%)	6 (16.2%)	3 (30.0%)
stage II	12 (10.6%)	4 (10.3%)	1 (2.7%)	0 (0.0%)
stage III	18 (15.9%)	6 (15.4%)	7 (18.9%)	1 (10.0%)
stage IV	54 (47.8%)	21 (53.8%)	17 (45.9%)	4 (40.0%)
**Radiotherapy**				
no	44 (40.0%)	12 (30.8%)	10 (27.0%)	5 (50.0%)
yes	69 (60.0%)	27 (69.2%)	27 (73.0%)	5 (50.0%)
**Chemotherapy**				

no	40 (35.4%)	6 (15.4%)	11 (29.7%)	4 (40.0%)
yes	73 (64.6%)	33 (84.6%)	26 (70.3%)	6 (60.0%)
**Survival status**				
live	81 (71.7%)	17 (43.6%)	16 (43.2%)	4 (40.0%)
dead	32 (28.3%)	22 (56.4%)	21 (56.8%)	6 (60.0%)
**Primary site**				
tongue	32 (28.3%)	--	--	--
cheek	34 (30.0%)	--	--	--
others	47 (41.6%)	--	--	--

Table S2. Clinical characteristics of the subjects in *GALNT14* mRNA expression analysis.

	HNSCC			
	**OSCC**	**OPSCC**	**LSCC**	**others**
**Mean of age**	56.67±11.72	58.65±8.054	58.8±12.08	55
Gender				
male	26	18	20	1
female	1	2	0	0
**Alcohol**				
no	13	4	6	0
yes	14	16	14	1
**Betel nut**				
no	12	4	9	0
yes	15	16	11	1
**Cigarette**				
no	8	2	4	0
yes	19	18	16	1
**Differentiation**				
no record	3	0	1	0
well	6	1	0	0
moderate	18	15	19	1
poor	0	4	0	0
**TNM**				
no record	6	1	6	0
T0	0	0	0	0
T1	4	2	9	0
T2	5	11	4	0
T3	1	2	0	0
T4	11	4	1	1
**TNM**				
no record	7	2	5	0
n=0	9	9	5	1
N=1	3	1	0	0
N=2	8	8	10	0
N=3	0	0	0	0
**Stage**				
no record	5		4	0
stage BBB	1	1	2	0
stage I	3	2	7	0
stage II	2	3	0	0
stage III	3	3	0	0
stage IV	13	11	7	1
**Radiotherapy- survival status**				
no	9	7	9	0
yes	18	13	11	1
**Chemotherapy- survival status**				
no	9	3	12	0
yes	18	17	8	1
**Survival status**				
live	12	7	8	0
dead	15	13	12	1

**Primary site**				
tongue	4	--	--	--
cheek	8	--	--	--
others	15	--	--	--
